# Clinical Characteristics and Survival Outcomes of Metastatic Invasive Lobular and Ductal Carcinoma

**DOI:** 10.1001/jamanetworkopen.2025.1888

**Published:** 2025-04-28

**Authors:** Akshara S. Raghavendra, Roland Bassett, Senthil Damodaran, Carlos H. Barcenas, Jason A. Mouabbi, Rachel Layman, Debu Tripathy

**Affiliations:** 1 Division of Cancer Medicine, Department of Breast Medical Oncology, The University of Texas MD Anderson Cancer Center, Houston; 2 Department of Biostatistics, The University of Texas MD Anderson Cancer Center, Houston

## Abstract

**Importance:**

Comparing the clinical and molecular features of metastatic invasive lobular carcinoma (mILC) and metastatic invasive ductal carcinoma (mIDC) is essential to enhance understanding of breast cancer biology and improve personalized treatment approaches.

**Objective:**

To compare mILC and mIDC in terms of survival outcomes and to investigate the association of clinicopathologic characteristics with those outcomes.

**Design, Setting, and Participants:**

This cohort study included adult patients at the University of Texas MD Anderson Cancer Center with their first metastatic diagnosis occurring between January 1997 and December 2020. Patient records were obtained from an institutional database. The study follow-up concluded in July 2023, and data were analyzed from July to December 2024.

**Exposure:**

Diagnosis of mIDC and mILC.

**Main Outcomes and Measures:**

Progression-free survival (PFS), overall survival (OS), and disease-free interval (DFI) were estimated using the Kaplan-Meier method. Survival distributions were compared using the log-rank test. Multivariable Cox proportional hazards regression was used to assess the association of metastasis onset, estrogen receptor (ER) expression level, and tumor grade with OS and PFS.

**Results:**

The analysis included a total of 9714 patients (9628 women [99%]), 8535 with mIDC and 1179 with mILC. The median age at metastasis was 53.3 years (range, 17.6-62.0 years). Generally, patients with mILC were older and had lower nuclear grade tumors and fewer metastasis sites than patients with mIDC. Patients with mILC had longer PFS (median, 0.65 years; 95% CI, 0.58-0.74) than patients with mIDC (median, 0.46 years; 95% CI, 0.45-0.48) (hazard ratio [HR], 0.78; 95% CI, 0.73-0.84; *P* < .001). For OS, patients with mILC had longer OS (median, 3.06 years; 95% CI, 2.87-3.29) than patients with mIDC (median, 2.60 years; 95% CI, 2.52-2.67 years) (HR, 0.91; 95% CI, 0.84-0.98; *P* = .01). Overall, patients with mILC had longer DFI than patients with mIDC (HR, 0.69; 95% CI, 0.64-0.75; *P* < .001). At initial diagnosis, patients with mILC were less likely to present with visceral metastasis (522 patients [44.3%]) than patients with mIDC (4909 patients [57.5%]). A higher proportion of patients with mILC (931 patients [79.0%]) than patients with mIDC (5431 patients [63.6%]) had bone-only metastasis.

**Conclusions and Relevance:**

In this cohort study, patients with mILC had longer OS and PFS than those with mIDC. Metastasis onset, ER positivity, and tumor grade were associated with survival outcomes, and distinct metastatic patterns of mIDC and mILC were also associated with survival for mIDC and mILC, which may help guide more personalized treatment strategies for each subtype.

## Key Points

**Question:**

How do the clinicopathological features and metastatic patterns of metastatic invasive lobular carcinoma (mILC) and metastatic invasive ductal carcinoma (mIDC) differ?

**Findings:**

In this cohort study of 9714 patients (8535 with mIDC and 1179 with mILC), those with mILC had longer progression-free survival (PFS) and overall survival (OS) than those with mIDC; estrogen receptor positivity and lower tumor grade were associated with longer PFS and OS. Patients with mILC had fewer visceral metastases and more bone-only metastasis.

**Meaning:**

These findings suggest that distinct metastatic patterns in mILC and mIDC are associated with survival outcomes, which may help guide more personalized treatment strategies for each subtype.

## Teaser

This cohort study examines the clinicopathological features and metastatic patterns of metastatic invasive lobular carcinoma and metastatic invasive ductal carcinoma and investigates their association with survival outcomes.

## Introduction

Breast cancer subtypes differ in biology, clinical behavior, and treatment response; the 2 most common subtypes are invasive ductal carcinoma (IDC) and invasive lobular carcinoma (ILC). IDC is the most common histologic subtype, accounting for 70% to 80% of all breast cancer cases.^1^ It predominantly affects postmenopausal women but can occur at any age. Risk factors for IDC include age, family history of breast cancer, germline pathogenic variants (such as *BRCA1* and *BRCA2* variants), hormonal factors (eg, early menarche and late menopause), and obesity. IDC often presents as a palpable breast mass with spiculated or irregular margins on mammography. In contrast, ILC accounts for 10% to 15% of all breast cancers.^2,3,4^ Patients with ILC tend to be older (perimenopausal and postmenopausal). ILC risk factors include family history of breast cancer and personal history of lobular carcinoma in situ.^5^ Unlike IDC, ILC often presents with subtle clinical findings and can be challenging to detect on mammography owing to its ill-defined margins and lack of architectural distortion.^6,7^

Metastatic ILC (mILC) and metastatic IDC (mIDC) have different clinicopathologic characteristics and responsiveness to systemic therapy.^8,9,10,11^ For example, mILC is often diagnosed at a later stage than mIDC, which can be attributed to the subtle nature of its growth pattern and the associated challenges of early detection with usual imaging techniques.^12^ Unlike mILC, mIDC often presents as a palpable mass, likely resulting in earlier diagnosis.^13^ Because mILC is rarer than mIDC, most clinical studies of mILC have had small patient cohorts.

We conducted a large study comparing the characteristics and outcomes of patients with mILC and mIDC. Our goal was to improve the current understanding of mILC and its differences from mIDC to facilitate clinical decision-making and uncover potential avenues for further studies.

## Methods

### Study Design

This retrospective cohort study analyzed mIDC and mILC cases from The University of Texas MD Anderson Cancer Center. Ethical approval for this study was obtained from MD Anderson’s institutional review board. A waiver of informed consent was obtained because the data were deidentified, in accordance with 45 CFR §46. This study adhered to the Strengthening the Reporting of Observational Studies in Epidemiology (STROBE) reporting guidelines.

### Patient Population

**Figure 1.**
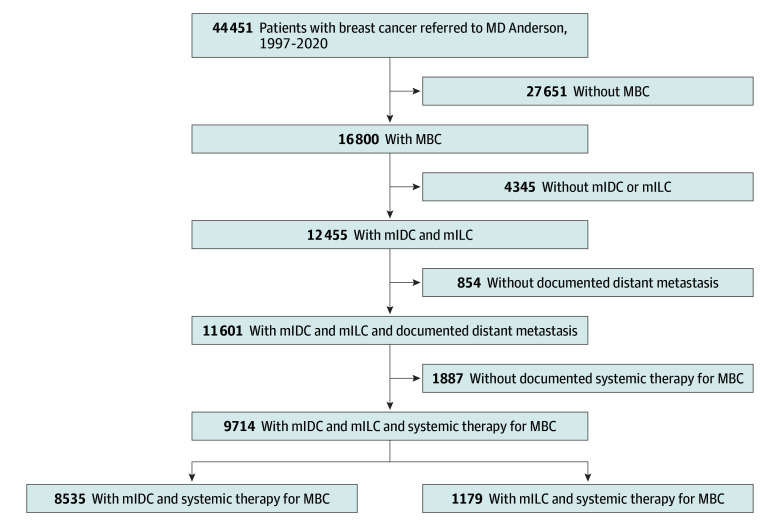
Patient Enrollment Flowchart MBC indicates metastatic breast cancer; mIDC, metastatic invasive ductal carcinoma; mILC, metastatic invasive lobular carcinoma.

To identify patients for inclusion, we searched the Department of Breast Medical Oncology’s Breast Cancer Research Database, an extensive clinical oncology database that contains a comprehensive record of patients with breast cancer treated at MD Anderson (at least 3 visits) since 1997. We included all adult patients with mILC or mIDC who were referred to MD Anderson between January 1997 and December 2020 and had distant recurrence after diagnosis of stage I to III breast cancer (recurrent metastases) or stage IV breast cancer at initial diagnosis (de novo metastases) (Figure 1). Patients who did not experience metastasis during the study period were excluded. The study follow-up concluded in July 2023, and data were analyzed from July to December 2024.

We collected patient demographic and clinicopathologic data (age, race, primary tumor stage, clinical nodal status, hormone receptor status, Ki67 levels, sites of metastasis, history of treatments for metastatic breast cancer, and disease status at the time of last contact or death) from the database. Race and ethnicity were self-reported by participants using predefined options provided in the electronic medical records: American Indian, Asian or Pacific Islander, Black, Hispanic or Spanish, White, and other, which includes Alaska Native and unknown. These options were developed to align with standard demographic categories. The assessment of race was included in the study to explore potential disparities in the study outcome. Hormone receptor positivity was defined on the basis of estrogen receptor (ER) expression or progesterone receptor positivity as determined by standard immunohistochemistry using institutional cutoffs adhering to the concurrent American Society of Clinical Oncology guidelines.^14^ ER positivity was categorized according to the proportion of cells with positive staining of tumor nuclei (0%-9%, 10%-55%, 56%-94%, and ≥95%). The ERBB2 (erb-b2 receptor tyrosine kinase 2; formerly HER2) status^15^ of the primary tumors was determined using either immunohistochemistry or gene amplification via fluorescence in situ hybridization. We categorized patients into mILC and mIDC groups. Patients were further subcategorized according to metastasis onset, ER expression level, and tumor grade.

The genomic testing data were sourced from a prospectively maintained database of molecular testing reports from Clinical Laboratory Improvement Amendments–certified laboratories, accessed under an approved institutional review board protocol with a waiver of informed consent. Tumor profiling was conducted at MD Anderson, Foundation Medicine, and other Clinical Laboratory Improvement Amendments–certified laboratories, focusing on somatic genomic alterations.

### Definitions

Overall survival (OS) was defined as the time from the start of first-line systemic therapy for metastatic breast cancer to death. Patients who remained alive were censored at their last follow-up date.

Progression-free survival (PFS) was defined as the time from the start of first-line systemic therapy for metastatic breast cancer to the end of first-line therapy or death (whichever was first). Patients who remained alive and receiving first-line therapy were censored at their last follow-up date.

Disease-free interval (DFI) was defined as the time from the initial diagnosis to first metastasis. DFI was measured only for patients with recurrent metastases; patients with de novo metastases were excluded from this analysis.

### Statistical Analysis

For continuous variables, data were summarized using standard descriptive statistics, including mean (SD), median (IQR), 95% CI, and range; Wilcoxon rank-sum tests were used to compare the distribution of data between histologic groups. For categorical variables, data were summarized using frequencies and percentages, and Fisher exact tests were used to compare the distribution of data between groups. The Kaplan-Meier method was used to estimate distributions of OS, PFS, and DFI. Survival distributions were compared using the log-rank test. Multivariable Cox proportional hazards regression was used to assess the association between covariates of interest and PFS and OS. Because treatment for breast cancer has changed over time, as a check, propensity score weighting was computed according to the year of diagnosis of first metastasis and was used to weight patients for the survival outcomes.^16^ Analyses were consistent with the unweighted results, which are presented. All statistical analyses were performed using R statistical software version 4.3.1 (R Project for Statistical Computing) and a significance level of 2-sided *P* < .05. No adjustments for multiple testing were made.

## Results

### Patient Characteristics

The study included 9714 patients with metastatic breast cancer (8535 with mIDC and 1179 with mILC; 9628 women [99%]). For all patients, the mean (SD) body mass index (calculated as weight in kilograms divided by height in meters squared) was 28.4 (6.8) (range, 13.8-88.9). The median age at first metastasis was 53.3 years (range, 17.6-97.3 years). Table 1 summarizes patient characteristics by histologic group. Patients with mILC were significantly older and had fewer metastases than patients with mIDC. Among women in the study, 8451 (99.0%) had mIDC and 1177 (99.8%) had mILC. Although men made up a small proportion of the cohort (86 patients [0.9%]), a majority had mIDC (84 patients [1.0%]) compared with mILC (2 patients [0.2%]).

**Table 1.** Patient Characteristics by Histologic Group

Characteristic	Patients, No. (%)		*P* value
	mIDC (n = 8535)	mILC (n = 1179)	
Body mass index, mean (SD)^a^	28.5 (6.8)	28.1 (6.5)	.17
Age at first metastasis, mean (SD), y	52.6 (12.3)	58.2 (11.6)	<.001
No. of metastases, mean (SD)	2.9 (1.8)	2.6 (1.6)	*<.*001
Sex			
Female	8451 (99.0)	1177 (99.8)	.002
Male	84 (1.0)	2 (0.2)	
Race and ethnicity			
American Indian	18 (0.2)	1 (0.1)	<.001
Asian or Pacific Islander	379 (4.4)	32 (2.7)	
Black	1116 (13.1)	95 (8.1)	
Spanish or Hispanic	888 (10.4)	120 (10.2)	
White	6024 (70.6)	916 (77.7)	
Other^b^	110 (1.3)	15 (1.3)	
Family history of breast or ovarian cancer			
No	6727 (78.8)	853 (72.3)	*<.*001
Yes	1808 (21.2)	326 (27.6)	
Site of metastasis at first diagnosis			
Nonvisceral	3626 (42.5)	657 (55.7)	*<.*001
Visceral	4909 (57.5)	522 (44.3)	
Timing of metastasis			
De novo	2491 (29.2)	409 (34.7)	*<.*001
Recurrent	6044 (70.8)	770 (65.3)	
Primary tumor cells expressing estrogen receptor, %			
0-9	2037 (28.8)	78 (7.9)	*<.*001
10-55	762 (10.8)	118 (12.0)	
56-94	2115 (29.9)	397 (40.2)	
≥95	2152 (30.5)	394 (39.9)	
Biomarker subtypes			
Hormone receptor positive, ERBB2 negative	4606 (54.0)	985 (83.5)	<.001
ERBB2 positive	1966 (23.0)	92 (7.8)	
Triple-negative breast cancer	1963 (23.0)	102 (8.6)	
Primary tumor cells expressing Ki67, %			
≤10	393 (12.3)	185 (42.0)	*<.*001
11-14	40 (1.2)	9 (2.0)	
15-20	471 (14.7)	87 (19.8)	
21-30	471 (14.7)	64 (14.6)	
*>*30	1827 (57.1)	95 (21.6)	
Unknown	5333^c^	739^c^	
Tumor grade			
1	150 (2.0)	186 (19.4)	*<.*001
2	2384 (32.1)	558 (58.1)	
3	4901 (65.9)	216 (22.5)	
Unknown	1100^c^	219^c^	

Abbreviations: ERBB2, erb-b2 receptor tyrosine kinase 2; mIDC, metastatic invasive ductal carcinoma; mILC, metastatic invasive lobular carcinoma.

^a^ Body mass index is calculated as weight in kilograms divided by height in meters squared.

^b^ Other includes Alaska Native and unknown race or ethnicity. The totals in some categories are less than the total number of included patients because of missing data.

^c^ Unknown values were not included in calculations of percentages.

The 2 histologic groups had similar racial and ethnic distributions. Among American Indian individuals, 18 (0.2%) had mIDC and 1 (0.1%) had mILC; among Asian and Pacific Islander patients, 379 (4.4%) had mIDC and 32 (2.7%) had mILC; among Black individuals, 1116 (13.1%) had mIDC and 95 (8.1%) had mILC; among Spanish or Hispanic individuals, 888 (10.4%) had mIDC and 120 (10.2%) had mILC; among White individuals, 6024 (70.6%) had mIDC and 916 (77.7%) had mILC; and among patients who identified as other race or ethnicity, 110 (1.3%) had mIDC and 15 (1.3%) had mILC.

A higher proportion of patients with mILC (326 patients [27.6%]) than patients with mIDC (1808 patients [21.2%]) had a family history of breast or ovarian cancer. The hormone receptor–positive and ERBB2-negative subtype was predominant in mILC (985 patients [83.5%]) vs mIDC (4606 patients [54.0%]). In contrast, ERBB2-positive (1966 patients [23.0%]) and triple-negative breast cancer (TNBC; 9631 patients [23.0%]) subtypes were much less frequent among mILC cancers. Patients with mILC had lower Ki67 levels than patients with mIDC. Grade 2 tumors were significantly more common among patients with mILC (558 patients [58.1%]) than among patients with mIDC (2384 patients [32.1%]), whereas grade 3 tumors were more prevalent among patients with mIDC (4901 patients [65.9%[) than among patients with mILC (216 patients [22.5%]). Although recurrent metastases were more common than de novo metastases in both histologic groups, patients with mILC (409 patients [34.7%]) were more likely than those with mIDC (2491 patients [29.2%]) to have de novo metastases.

**Figure 2.**
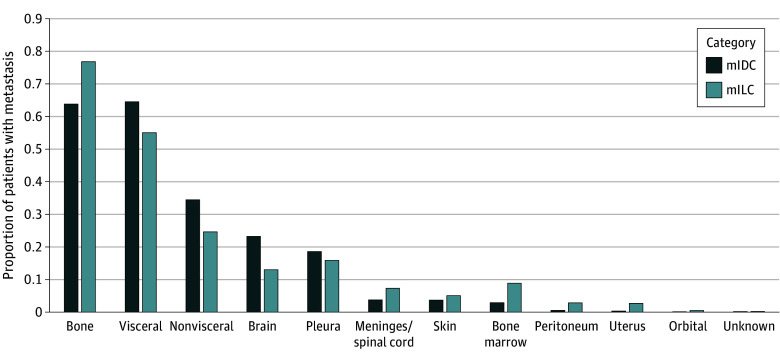
Proportion of Patients With Metastases When biopsy findings were unavailable, bone marrow involvement was determined on the basis of blood count results and morphologic findings. mIDC indicates metastatic invasive ductal carcinoma; mILC, metastatic invasive lobular carcinoma.

Fewer patients with mILC (522 patients [44.3%]) than patients with mIDC (4909 patients [57.5%]) presented with visceral metastasis during the entire disease course and at initial diagnosis (eFigures 1 and 2 in Supplement 1). Metastases in the bone, meninges, bone marrow, peritoneum, and uterus were significantly more prevalent in patients with mILC than in patients with mIDC (Figure 2). Conversely, more patients with mIDC than patients with mILC had metastases in visceral and nonvisceral sites, the brain, and the pleura. The proportion of patients with only nonbone metastasis was higher among patients with mIDC (3104 patients [36.4%]) than among patients with mILC (248 patients [21.0%]) during the entire disease course, whereas bone-only metastasis was more common in patients with mILC (285 patients [24.2%]) than among patients with mIDC (1322 patients [15.5%]). At the first distant metastasis site, bone-only metastasis was more common among patients with mILC (608 patients [51.6%]) than among those with mILC (2850 patients [33.4%]) (eFigures 3 and 4 in Supplement 1).

### Progression-Free Survival

The median PFS for all patients was 0.48 years (95% CI, 0.47-0.50). Hazard ratios (HRs) reflect mILC vs mIDC; thus, HRs less than 1 reflect a lower hazard for mILC and, thus, better PFS. Patients with mILC had significantly longer PFS (median, 0.65 years; 95% CI, 0.58-0.74 years) than patients with mIDC (median, 0.46 years; 95% CI, 0.45-0.48 years) (HR, 0.78; 95% CI, 0.73-0.84; *P* < .001). For patients with mIDC, the 1-year PFS rate was 26.6% (95% CI, 25.6%-27.6%), and the 5-year PFS rate was 3.1% (95% CI, 2.7%-3.6%). For patients with mILC, the 1-year PFS rate was 39.4% (95% CI, 36.5%-42.4%), and the 5-year PFS rate was 5.3% (95% CI, 4.0%-7.1%) (eFigure 5A in Supplement 1). Table 2 and eFigure 6 in Supplement 1 present the HRs for PFS between mILC and mIDC according to metastasis onset, ER expression level, and tumor grade. Multivariable analysis showed better prognosis in mILC than mIDC (HR, 0.92; 95% CI, 0.85-1.00; *P* = .049) (eTable 1 in Supplement 1).

**Table 2.** PFS for Each Histologic Group by Timing of Metastasis, Estrogen Receptor Expression, and Tumor Grade

Variable	mIDC		mILC		HR (95% CI)	*P* value
	Patients, No.	PFS, median (95% CI), y	Patients, No.	PFS, median (95% CI), y		
Histologic group	7851	0.47 (0.45-0.48)	1089	0.63 (0.56-0.70)	0.78 (0.73-0.84)	<.001
Timing of metastasis						
De novo	2407	0.45 (0.43-0.47)	389	0.57 (0.50-0.75)	0.76 (0.68-0.86)	<.001
Recurrent	5444	0.48 (0.46-0.50)	700	0.67 (0.60-0.76)	0.79 (0.73-0.86)	<.001
Subtype						
Hormone receptor positive and ERBB2 negative	4256	0.53 (0.50-0.55)	913	0.69 (0.62-0.79)	0.82 (0.76-0.89)	<.001
ERBB2 positive	1822	0.57 (0.52-0.61)	85	0.60 (0.50-0.91)	0.91 (0.73-1.14)	.41
Triple-negative breast cancer	1773	0.33 (0.33-0.34)	91	0.34 (0.27-0.46)	0.70 (0.57-0.88)	<.001
Primary tumor cells expressing estrogen receptor, %						
0-9	1868	0.38 (0.36-0.40)	70	0.34 (0.27-0.46)	1.00 (0.78-1.27)	.99
10-55	695	0.42 (0.40-0.46)	106	0.76 (0.59-1.16)	0.71 (0.58-0.87)	.001
56-94	1967	0.54 (0.50-0.57)	368	0.75 (0.63-0.86)	0.81 (0.72-0.91)	<.001
≥95	2002	0.64 (0.59-0.69)	367	0.67 (0.56-0.97)	0.93 (0.83-1.05)	.25
Tumor grade						
1	141	0.64 (0.52-1.16)	177	0.75 (0.59-1.05)	0.91 (0.72-1.16)	.45
2	2220	0.59 (0.56-0.65)	510	0.70 (0.59-0.87)	0.93 (0.84-1.03)	.15
3	4485	0.42 (0.41-0.42)	199	0.50 (0.42-0.58)	0.86 (0.74-0.99)	.04

Abbreviations: ERBB2, erb-b2 receptor tyrosine kinase 2; HR, hazard ratio; mIDC, metastatic invasive ductal carcinoma; mILC, metastatic invasive lobular carcinoma; PFS, progression-free survival.

#### Metastasis Onset

In the mIDC group, the median PFS was similar in patients with de novo metastases (0.45 years; 95% CI, 0.43-0.47 years) and those with recurrent metastases (0.48 years; 95% CI, 0.46-0.50 years), whereas in the mILC group, the median PFS was shorter in patients with de novo metastases (0.57 years; 95% CI, 0.50-0.75 years) than in those with recurrent metastases (0.67 years; 95% CI, 0.60-0.76 years) (Table 2). In both metastasis-onset groups, patients with mILC had longer PFS than patients with mIDC (eFigure 5B in Supplement 1).

#### Biomarker Subtypes

In patients with the hormone receptor–positive and ERBB2-negative subtype, the mILC group demonstrated better PFS compared with the mIDC group. At 1 year, patients with mILC had a PFS of 41.0% (95% CI, 37.9%-44.4%) vs 30.5% (95% CI, 29.1%-31.9%) for patients with mIDC, and at 5 years, patients with mILC had a PFS of 5.4% (95% CI, 3.9%-7.4%) vs 3.5% (95% CI, 2.9%-4.2%) (*P* < .001) for mIDC. In patients with the ERBB2-positive subtype, no significant difference in PFS was observed between mILC and mIDC histologic types. At 1 year, patients with mILC had a PFS of 35.3% (95% CI, 26.3%-47.4%) vs 32.8% (95% CI, 30.7%-35.0%) for mIDC, and at 5 years, patients with mILC had PFS of 7.0% (95% CI, 3.1%-15.9%) vs 4.4% (95% CI, 3.5%-5.6%) for mIDC (*P* = .41). Among patients with TNBC, those with mILC had better PFS than those with mIDC. At 1 year, patients with mILC had a PFS of 26.4% (95% CI, 18.6%-37.3%) vs 11.0% (95% CI, 9.6%-12.6%) for mIDC, and at 5 years, patients with mILC had PFS of 2.6% (95% CI, 0.7%-10.1%) vs 0.7% (95% CI, 0.3%-1.3%) for those with mIDC (*P* = .002). In addition, across all subtypes, PFS rates were highest in the group who received a metastatic diagnosis in the years 2016 to 2020, with 1-year PFS of 28.7% (95% CI, 26.5%-31.1%) compared with 18.2% (95% CI, 15.2%-21.6%) in the 1993 to 2000 cohort (*P* < .001).

#### Tumor Grade

In both histologic groups, the median PFS decreased as tumor grade increased (Table 2 and eFigure 5D in Supplement 1). Across all tumor grades, patients with mILC had longer median PFS than patients with mIDC. Patients with mIDC and mILC had similar PFS for grade 1 (HR, 0.91; 95% CI, 0.72-1.16; *P* = .45) and grade 2 (HR, 0.93; 95% CI, 0.84-1.03; *P* = .15) tumors. For grade 3 tumors, patients with mILC had better PFS than patients with mIDC (HR, 0.86; 95% CI, 0.74-0.99; *P* = .04).

### Overall Survival

The median OS for all patients was 2.66 years (95% CI, 2.59-2.73 years). Patients with mILC had longer survival (median, 3.06 years; 95% CI, 2.87-3.29 years) than patients with mIDC (median, 2.60 years; 95% CI, 2.52-2.67 years) (HR, 0.91; 95% CI, 0.84-0.98; *P* = .01) (Table 3). For mIDC, the 1-year OS rate was 79.5% (95% CI, 78.6%-80.4%), the 5-year rate was 25.5% (95% CI, 24.4%-26.7%), and the 15-year rate was 5.4% (95% CI, 4.6%-6.4%); for mILC, the 1-year OS rate was 85.4% (95% CI, 83.3%-87.6%), the 5-year rate was 28.4% (95% CI, 25.4%-31.7%), and the 15-year rate was 3.3% (95% CI, 1.7%-6.6%) (eFigure 7A in Supplement 1). Table 3 and eFigure 8 in Supplement 1 show the HRs for OS between mILC and mIDC according to metastasis onset, ER expression, and tumor grade.

**Table 3.** OS for Each Histologic Group by Timing of Metastasis, Estrogen Receptor Expression, and Tumor Grade

Variable	mIDC		mILC		HR (95% CI)	*P* value
	Patients, No.	OS, median (95% CI), y	Patients, No.	OS, median (95% CI), y		
Histologic group	7851	2.60 (2.52-2.67)	1089	3.05 (2.89-3.27)	0.91 (0.84-0.98)	.01
Timing of metastasis						
De novo	2407	3.62 (3.39-3.77)	389	3.87 (3.39-4.36)	0.91 (0.79-1.04)	.16
Recurrent	5444	2.20 (2.12-2.31)	700	2.60 (2.46-2.87)	0.94 (0.86-1.03)	.18
Subtype						
Hormone receptor positive and ERBB2 negative	4256	3.05 (2.94-3.18)	913	3.04 (2.87-3.27)	1.01 (0.93-1.10)	.80
ERBB2 positive	1822	3.35 (3.16-3.57)	85	3.96 (3.36-5.54)	0.87 (0.66-1.15)	.33
Triple-negative breast cancer	1773	1.22 (1.17-1.28)	91	1.85 (1.22-3.33)	0.68 (0.53-0.86)	<.001
Primary tumor cells expressing estrogen receptor, %						
0-9	1868	1.48 (1.40-1.58)	70	1.49 (1.02-2.50)	0.99 (0.76-1.30)	.97
10-55	695	2.24 (2.06-2.54)	106	3.24 (2.72-3.70)	0.85 (0.67-1.07)	.15
56-94	1967	3.14 (3.01-3.35)	368	3.02 (2.74-3.40)	1.04 (0.91-1.19)	.56
≥95	2002	3.80 (3.61-4.05)	367	3.12 (2.85-3.54)	1.25 (1.09-1.44)	.002
Tumor grade						
1	141	4.47 (4.04-5.99)	177	3.27 (2.86-3.87)	1.31 (1.04-1.87)	.03
2	2220	3.57 (3.38-3.74)	510	3.12 (2.87-3.39)	1.16 (1.03-1.30)	.02
3	4485	1.98 (1.90-2.06)	199	2.04 (1.84-2.72)	0.97 (0.83-1.14)	.74

Abbreviations: ERBB2, erb-b2 receptor tyrosine kinase 2; HR, hazard ratio; mIDC, metastatic invasive ductal carcinoma; mILC, metastatic invasive lobular carcinoma; OS, overall survival.

#### Metastasis Onset

In both histologic groups, patients with de novo metastases had longer median OS than those with recurrent metastases (Table 3). Across both metastasis-onset groups, patients with mILC had slightly longer median OS than patients with mIDC (eFigure 7B in Supplement 1), although comparisons were not statistically significant.

#### Biomarker Subtypes

In patients with the hormone receptor–positive, ERBB2-negative subtype, no significant difference in OS was observed between the mILC group and the mIDC group. At 1 year, patients with mILC had an OS of 87.2% (95% CI, 85.0%-89.5%) compared with 84.9% (95% CI, 83.8%-86.0%) for those with mIDC, and at 5 years, patients with mILC had OS of 27.2% (95% CI, 24.0%-30.8%) vs 28.1% (95% CI, 26.6%-29.8%) for those with mIDC (*P* = .80). In patients with ERBB2-positive cancer, no significant difference in OS was observed between mILC and mIDC histologic subtypes. At 1 year, patients with mILC had an OS of 87.7% (95% CI, 80.8%-95.1%) vs 87.5% (95% CI, 86.0%-89.1%) for those with mIDC, and at 5 years, patients with mILC had OS of 47.5% (95% CI, 37.3%-60.6%) vs 34.8% (95% CI, 32.4%-37.4%) for those with mIDC (*P* = .34). In patients with TNBC, those with mILC exhibited significantly better OS than those with mIDC. At 1 year, patients with mILC had an OS of 65.4% (95% CI, 56.1%-76.3%) vs 58.4% (95% CI, 56.1%-60.8%) among those with mIDC, and at 5 years, patients with mILC had OS of 21.6% (95% CI, 13.8%-33.8%) vs 9.7% (95% CI, 8.3%-11.4%) among those with mIDC (*P* = .002). In addition, across all subtypes, OS rates were highest in the group who received a metastatic diagnosis from 2016 to 2020, with 1-year OS of 77.9% (95% CI, 75.7%-80.1%) compared with 75.1% (95% CI, 71.6%-78.8%) in the 1993 to 2000 cohort (*P* < .001).

#### Tumor Grade

Higher tumor grade was generally associated with lower OS for both histologic groups (eFigure 7D in Supplement 1). For patients with grade 1 tumors, OS was longer for those with mIDC than for those with mILC (HR, 1.39; 95% CI, 1.04-1.87; *P* = .03). For patients with grade 2 tumors, OS was significantly longer for patients with mIDC (HR, 1.16; 95% CI, 1.03-1.30; *P* = .02). For patients with grade 3 tumors, OS was similar between mILC and mIDC (HR, 0.97; 95% CI, 0.83-1.14; *P* = .74) (Table 3).

From the multivariable analysis depicted in eTable 1 in Supplement 1, patients with mILC demonstrated slightly better PFS compared with those with mIDC (HR, 0.92; 95% CI, 0.85-1.00; *P* = .049). However, mILC was associated with worse OS compared with mIDC (HR, 1.15; 95% CI, 1.05-1.26; *P* = .003). These findings may reflect the influence of correlated covariates and interactions among prognostic factors, which can contribute to instability in the survival estimates.

Because patients who received diagnoses in different years may have had different prognoses according to the treatments available, as a secondary analysis, propensity score weighting was used to control for bias in the comparison of mIDC and patients with mILC. Weights were computed on the basis of the year of diagnosis of first metastasis and were used to weight patients for the survival outcomes. Results were generally consistent with the unweighted results, which are presented.

### Disease-Free Interval

The median DFI for all patients was 2.58 years. Patients with mIDC had a shorter DFI (median, 2.47 years; 95% CI, 2.39-2.53 years) than patients with mILC (median, 3.93 years; 95% CI, 3.56-4.26 years) (HR, 0.69; 95% CI, 0.64-0.75; *P* < .001) (eFigure 9A and eTable 2 in Supplement 1). eTable 2 and eFigure 10 in Supplement 1 show the HRs for DFI between mILC and mIDC according to ER expression and tumor grade.

#### Subtype

In both histologic groups, hormone receptor–positive, ERBB2-negative subtypes exhibited the longest DFI (mIDC, median, 3.31 years; 95% CI, 3.21-3.43 years; mILC, median, 4.14 years; 95% CI, 3.81-4.47 years; HR, 0.83; 95% CI, 0.77-0.91; *P* < .001), whereas TNBC subtypes had the shortest DFI (mIDC, median, 1.53 years; 95% CI, 1.47-1.60 years; mILC, median, 1.59 years; 95% CI, 1.25-2.25 years; HR, 0.68; 95% CI, 0.51-0.91; *P* < .001). Estimates by subgroup can be found in (eFigure 9B and eTable 2 in Supplement 1).

#### Tumor Grade

In both histologic groups, tumor grade was inversely associated with DFI (eFigure 9C in Supplement 1). For patients with grade 1 tumors, the median DFI was longer for patients with mIDC than patients with mILC; for patients with grade 2 and 3 tumors, median DFI was longer for patients with mILC than patients with mIDC. Estimates can be found in eTable 2 in Supplement 1.

### Genomic Alterations

eFigure 11 and eTable 3 in Supplement 1 highlight the prevalence of specific genomic alterations in mIDC and patients with mILC across hormone receptor–positive and ERBB2-negative, ERBB2-positive, and TNBC subtypes. Notable alterations included *AKT1, CCND1–3, CCNE1*, *ERBB2, ESR1, FGFR1–4*, *GATA3, NF1, PIK3CA, RB1,* and *TP53* alterations.

In the hormone receptor–positive and ERBB2-negative subtype, patients with mIDC had higher rates of *TP53* variants (294 patients [21.9%] vs 40 patients [13.9%]), *GATA3* variants (49 patients [3.7%] vs 1 patient [0.3%]), and *FGFR* amplifications (79 patients [5.9%] vs 8 patients [2.8%]). On the other hand, patients with mILC had significantly higher rates of *PIK3CA* variants (109 patients [37.9%] vs 362 patients [27.0%]) and *NF1* variants (19 patients [6.6%] vs 40 patients [3.0%]). *ERBB2* variants were more frequent in mILC compared with mIDC (18 patients [6.2%] vs 33 patients [2.5%]). In the TNBC subtype, patients with mIDC had higher rates of *TP53* variants (234 patients [51.0%] vs 1 patient [4.8%]), and patients with mILC had a higher prevalence of *PIK3CA* mutations (5 patients [23.8%] vs 59 patients [12.8%]), although the difference was not statistically significant.

## Discussion

In this cohort study, we found that patients with mILC had longer PFS, OS, and DFI than patients with mIDC. The median PFS for all patients was 0.48 years; patients with mILC exhibited a slightly longer median PFS (0.65 years) than patients with mIDC (0.46 years). The median OS for all patients was 2.66 years, with patients with mILC having a slightly longer median OS (3.06 years) than patients with mIDC (2.60 years). Patients with mILC had better prognosis in the early-stage setting, having a slightly longer median DFI (3.93 years) than patients with mIDC (2.47 years). Compared with patients with mIDC, patients with mILC with de novo metastasis, higher ER positivity, and/or lower tumor grade had better PFS and OS estimates across 15 years, suggesting that long-term prognosis and survival patterns differ between patients with mILC vs those with mIDC.

A previous study^7^ emphasized that positive hormone receptor status, larger tumor size, higher histologic grade, and increased lymph node involvement were associated with slightly poorer prognosis in patients with mIDC than in patients with mILC. These factors also were associated with survival differently in mILC and patients with mIDC; the impacts on prognosis are unclear, with both better^17^ and worse^18^ outcomes having been observed.

Breast cancer study group trials have reported that patients with mILC have a better 5-year disease-free survival and OS than patients with mIDC,^19^ findings that were also observed in our study. In one study,^20^ patients with mILC had better survival rates during the first 5 years after diagnosis than patients with mIDC; however, survival significantly decreased after 10 to 15 years for patients with mILC. This trend was also seen in our study, emphasizing the importance of long-term follow-up and surveillance for these patients.^21^ We also found that late recurrences with longer DFI are more common in patients with mILC, a finding supported by previous studies, highlighting the need for ongoing monitoring past the initial treatment period.^22,23^

ER-positive, hormone receptor–positive tumors are more common in mILC than in mIDC,^10,24^ as observed in our study, and similarly, mILC tends to have a lower incidence of ERBB2 positivity than mIDC.^25^ In addition, mILC has different metastasis patterns than mIDC,^24,26,27^ which include a higher propensity for metastasis to the peritoneum, gastrointestinal tract, and ovary.^28^ In our study, bone-only metastasis was more common in mILC than in mIDC, and uncommon metastasis sites were mainly nonvisceral. Atypical metastatic dissemination has been documented in mILC cases; this pattern has been attributed to the loss of the adhesive protein E-cadherin, a defining feature of ILC.^29^ In mIDC, metastases to regional lymph nodes and distant organs, such as the lungs, liver, and bones, are common.^30^

We also investigated the genomic differences between mIDC and mILC tumors, as genomic alterations can affect outcomes and therapeutic responses. Several studies^31,32,33^ have identified genetic differences and variants specific to mILC. Notably, mILC exhibits genomic alterations, such as those in *FOXA1* and *GATA3*, and molecular signatures that differentiate it from mIDC^8,34^ and affect disease biology and treatment response.^33^ These genetic alterations help regulate hormone receptor signaling and mammary gland development.^35^

Furthermore, mILC has lower protein translation rates and overall metabolism than mIDC^36,37^; these differences may potentially influence PFS and explain why patients with mILC had better PFS than patients with mIDC. These differences in cellular metabolism between mIDC and mILC might have implications for their responses to targeted therapies and provide opportunities for developing subtype-specific treatment strategies.^38^

### Limitations and Strengths

Our study’s limitations include those posed by its retrospective design and inclusion of patients receiving treatment at outside institutions, which may have led to referral bias. To account for this bias, we included only individuals who had multiple visits at MD Anderson. A main strength of our study is that its use of a highly annotated and curated database from a single tertiary center enabled us to access robust patient data and extensive long-term follow-up information to calculate survival estimates up to 15 years.

## Conclusions

In this cohort study of patients with mIDC and mILC, metastasis onset, ER positivity, and tumor grade were associated with survival outcomes and the metastasis patterns of mIDC and mILC. The findings of the present study highlight the importance of recognizing the distinct prognostic factors of mIDC and mILC, which can improve our understanding of the association with disease progression, recurrence patterns, and survival outcomes, ultimately guiding more personalized treatment strategies for each subtype.

## Notes

Supplement 1. **eTable 1.** Multivariate Model Results for Progression-Free Survival (PFS) and Overall Survival (OS) **eTable 2.** Disease-Free Interval (DFI) for Each Histologic Group by Estrogen Receptor (ER) Expression and Tumor Grade **eTable 3.** Key Genomic Alterations in Patients With Metastatic Invasive Ductal Carcinoma (mIDC) and Metastatic Invasive Lobular Carcinoma (mILC) **eFigure 1.** Types of All Visceral Metastases **eFigure 2.** Types of Visceral Metastases at Dx **eFigure 3.** Types of All Metastases **eFigure 4.** Types of Metastasis at Dx **eFigure 5.** Progression-Free Survival **eFigure 6.** Forest Plot for PFS **eFigure 7.** Overall Survival **eFigure 8.** Forest Plot for OS **eFigure 9.** Disease-Free Interval **eFigure 10.** Forest Plot for DFI **eFigure 11.** Distribution of Key Genomic Alterations According to Histologic Type Across Subtypes

Supplement 2.
Data Sharing Statement
