# Supplementary material for: Clinical Characteristics and Survival Outcomes of Metastatic Invasive Lobular and Ductal Carcinoma
Source: JAMA Netw Open. 2025 Apr 28;8(4):e251888. doi: 10.1001/jamanetworkopen.2025.1888 (PMC12038507; doi:10.1001/jamanetworkopen.2025.1888)
Supplement: Supplement 1. — eTable 1. Multivariate Model Results for Progression-Free Survival (PFS) and Overall Survival (OS) eTable 2. Disease-Free Interval (DFI) for Each Histologic Group by Estrogen Receptor (ER) Expression and Tumor Grade eTable 3. Key Genomic Alterations in Patients With Metastatic Invasive Ductal Carcinoma (mIDC) and Metastatic Invasive Lobular Carcinoma (mILC) eFigure 1. Types of All Visceral Metastases eFigure 2. Types of Visceral Metastases at Dx eFigure 3. Types of All Metastases eFigure 4. Types of Metastasis at Dx eFigure 5. Progression-Free Survival eFigure 6. Forest Plot for PFS eFigure 7. Overall Survival eFigure 8. Forest Plot for OS eFigure 9. Disease-Free Interval eFigure 10. Forest Plot for DFI eFigure 11. Distribution of Key Genomic Alterations According to Histologic Type Across Subtypes [file jamanetwopen-e251888-s001.pdf]

Supplemental Online Content

Raghavendra AS, .Bassett R Jr, Damodaran S, et al. Clinical characteristics and survival outcomes of metastatic invasive lobular and ductal carcinoma. *JAMA Netw Open*. 2025;8(3):e251888. doi:10.1001/jamanetworkopen.2025.1888

eTable 1. Multivariate Model Results for Progression-Free Survival (PFS) and Overall Survival (OS)
eTable 2. Disease-Free Interval (DFI) for Each Histologic Group by Estrogen Receptor (ER) Expression and Tumor Grade
eTable 3. Key Genomic Alterations in Patients With Metastatic Invasive Ductal Carcinoma (mIDC) and Metastatic Invasive Lobular Carcinoma (mILC)
eFigure 1. Types of All Visceral Metastases
eFigure 2. Types of Visceral Metastases at Dx
eFigure 3. Types of All Metastases
eFigure 4. Types of Metastasis at Dx
eFigure 5. Progression-Free Survival
eFigure 6. Forest Plot for PFS
eFigure 7. Overall Survival
eFigure 8. Forest Plot for OS
eFigure 9. Disease-Free Interval
eFigure 10. Forest Plot for DFI
eFigure 11. Distribution of Key Genomic Alterations According to Histologic Type Across Subtypes

This supplemental material has been provided by the authors to give readers additional information about their work.

© 2025 Raghavendra AS et al. *JAMA Network Open*.

eTable 1: Multivariate Model Results for Progression-Free Survival (PFS) and Overall Survival (OS)

		PFS		OS	
**Variable**	**Level**	**HR (95% CI)**	**p-value**	**HR (95% CI)**	**p-value**
**Histology**	Ductal		.049		.003
	Lobular	0.92 (0.85-1.00)		1.15 (1.05-1.26)	
**Age at First Metastasis**	Continuous	1.00 (0.99-1.00)	<.001	1.01 (1.00-1.01)	<.001
**Number of Metastases**	Continuous	1.08 (1.07-1.09)	<.001	1.08 (1.07-1.10)	<.001
**Race**	ASIAN/PACIFIC IS		.001		<.001
	BLACK	1.13 (0.99-1.30)		1.93 (1.63-2.30)	
	NATIVE AMERICAN	1.62 (0.91-2.89)		1.02 (0.48-2.17)	
	OTHER	1.22 (0.97-1.53)		1.43 (1.04-1.96)	
	SPANISH, HISPANIC	1.08 (0.94-1.23)		1.43 (1.20-1.70)	
	WHITE	0.99 (0.88-1.12)		1.41 (1.21-1.66)	
**Site of Distant Metastasis**	Non-visceral		<.001		<.001
	Visceral	1.15 (1.09-1.20)		1.43 (1.36-1.51)	
**Timing of Metastasis**	De Novo		.006		<.001
	Recurrent	0.93 (0.89-0.98)		1.56 (1.47-1.65)	
**Subtype**	HR+/HER2-		<.001		<.001
	HER2+	0.75 (0.70-0.79)		0.69 (0.64-0.74)	
	TNBC	1.60 (1.50-1.71)		1.77 (1.65-1.89)	
**Nuclear Grade**	I		<.001		<.001
	II	1.19 (1.04-1.35)		1.09 (0.94-1.27)	
	III	1.54 (1.35-1.76)		1.56 (1.34-1.82)	

© 2025 Raghavendra AS et al. *JAMA Network Open*.

eTable2: Disease-free interval (DFI) for each histologic group by estrogen receptor (ER) expression and tumor grade.

	mIDC		mILC			
	**N**	**Median DFI (95% CI)**	**N**	**Median DFI (95% CI)**	**Hazard Ratio**	***P***
Histologic group	6044	2.47 (2.39‒2.55)	770	3.93 (3.56‒4.26)	0.69	<.001
Subtype						
HR+/HER2-	3306	3.31 (3.21, 3.43)	672	4.14 (3.81, 4.47)	0.83	<.001
HER2+	1257	2.12 (1.99, 2.28)	48	3.01 (2.14, 4.32)	0.68	.005
TNBC	1481	1.53 (1.47, 1.60)	50	1.59 (1.25, 2.25)	0.68	.005
Tumor grade						
1	91	5.39 (4.31‒6.63)	115	4.29 (3.74‒5.57)	1.10	.51
2	1609	3.52 (3.38‒3.66)	367	4.14 (3.64‒4.60)	0.89	.046
3	3612	1.95 (1.89‒2.02)	158	2.81 (2.03‒3.07)	0.77	<.001

CI, confidence interval; mIDC, metastatic invasive ductal carcinoma; mILC, metastatic invasive lobular carcinoma.

© 2025 Raghavendra AS et al. *JAMA Network Open*.

eTable 3. Key genomic alterations in patients with metastatic invasive ductal carcinoma (mIDC) and metastatic invasive lobular carcinoma (mILC) across subtypes.

Genomic Alteration	HR+HER2- mIDC, n (%)	HR+HER2- mILC, n (%)	*HR+HER2- p-value*	HER2+ mIDC, n (%)	HER2+ mILC, n (%)	HER2+ p-value	*TNBC mIDC, n (%)*	TNBC mILC, n (%)	TNBC p-value
**Mutation**									
TP53	294 (21.9%)	40 (13.9%)	.002	103 (27.2%)	4 (19.1%)	.61	234 (51.0%)	1 (4.8%)	<.001
PIK3CA	362 (27.0%)	109 (37.9%)	<.001	60 (15.8%)	4 (19.1%)	.76	59 (12.8%)	5 (23.8%)	.18
ESR1	137 (10.2%)	29 (10.1%)	1.00	13 (3.4%)	0 (0.0%)	1.00	9 (2.0%)	1 (4.8%)	.36
NF1	40 (3.0%)	19 (6.6%)	.005	9 (2.4%)	2 (9.5%)	.11	15 (3.3%)	1 (4.8%)	.52
FGFR	51 (3.8%)	12 (4.2%)	.74	6 (1.6%)	0 (0.0%)	1.00	12 (2.6%)	1 (4.8%)	.45
RB1	35 (2.6%)	10 (3.5%)	.43	7 (1.8%)	0 (0.0%)	1.00	22 (4.8%)	1 (4.8%)	1.00
ERBB2	33 (2.5%)	18 (6.2%)	.002	6 (1.6%)	4 (19.1%)	<.001	9 (2.0%)	0 (0.0%)	1.00
AKT1	32 (2.4%)	7 (2.4%)	1.00	8 (2.1%)	0 (0.0%)	1.00	9 (2.0%)	0 (0.0%)	1.00
GATA3	49 (3.7%)	1 (0.3%)	.001	7 (1.8%)	1 (4.8%)	0.35	3 (0.6%)	0 (0.0%)	1.00
Cyclin E	9 (0.7%)	1 (0.3%)	1.00	6 (1.6%)	0 (0.0%)	1.00	17 (3.7%)	0 (0.0%)	1.00
Cyclin D	22 (1.6%)	2 (0.7%)	.29	2 (0.5%)	0 (0.0%)	1.00	3 (0.6%)	0 (0.0%)	1.00
**Amplification**									
Cyclin D	104 (7.8%)	17 (5.9%)	.32	17 (4.5%)	1 (4.8%)	1.00	20 (4.4%)	0 (0.0%)	1.00
FGFR	79 (5.9%)	8 (2.8%)	.03	9 (2.4%)	0 (0.0%)	1.00	20 (4.4%)	0 (0.0%)	1.00

© 2025 Raghavendra AS et al. *JAMA Network Open*.

eFigure 1: Types of All Visceral Metastases

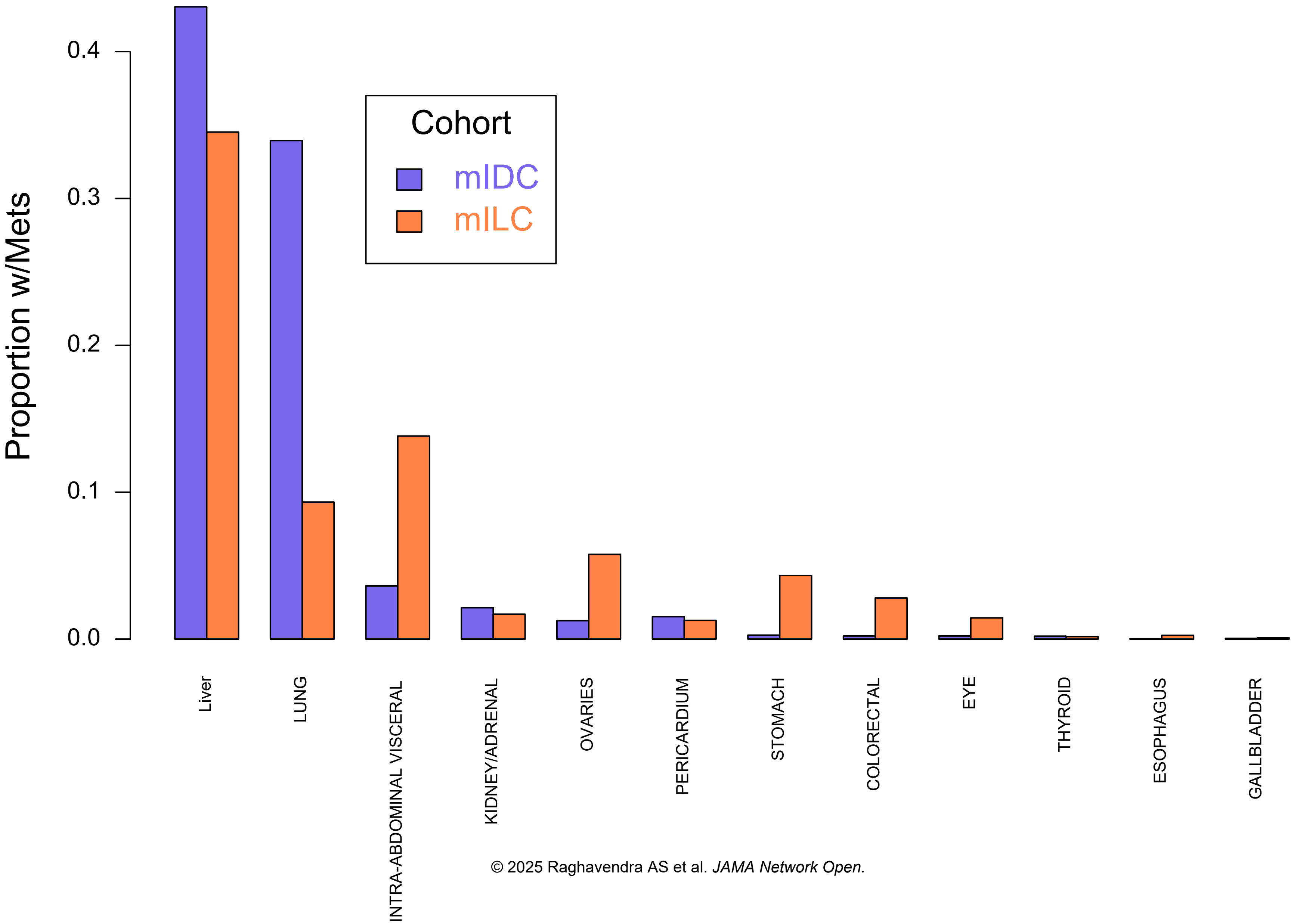

© 2025 Raghavendra AS et al. *JAMA Network Open.*

eFigure 2: Types of Visceral Metastases at Dx

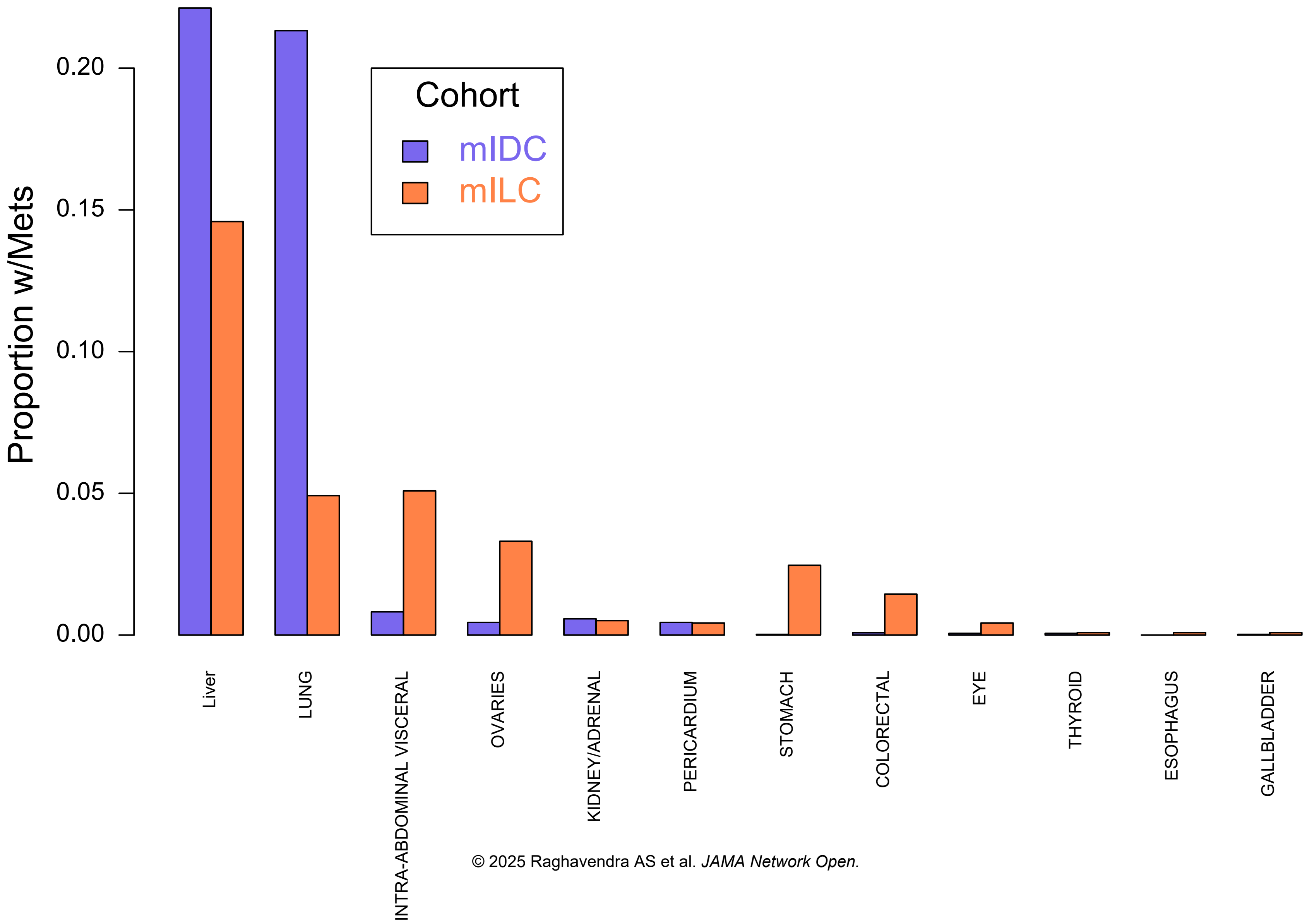

© 2025 Raghavendra AS et al. *JAMA Network Open.*

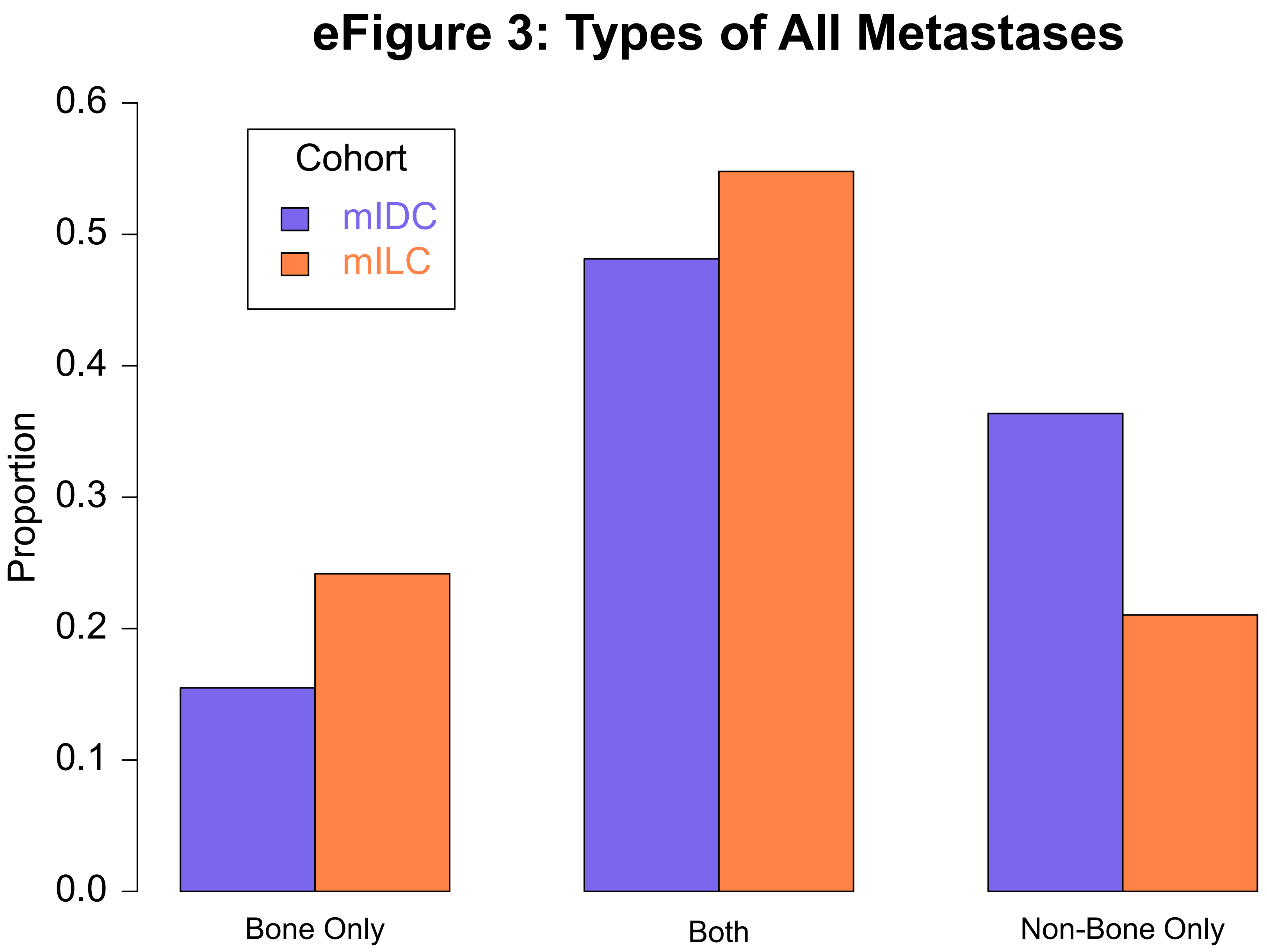

© 2025 Raghavendra AS et al. *JAMA Network Open.*

eFigure 4: Types of Metastasis at Dx

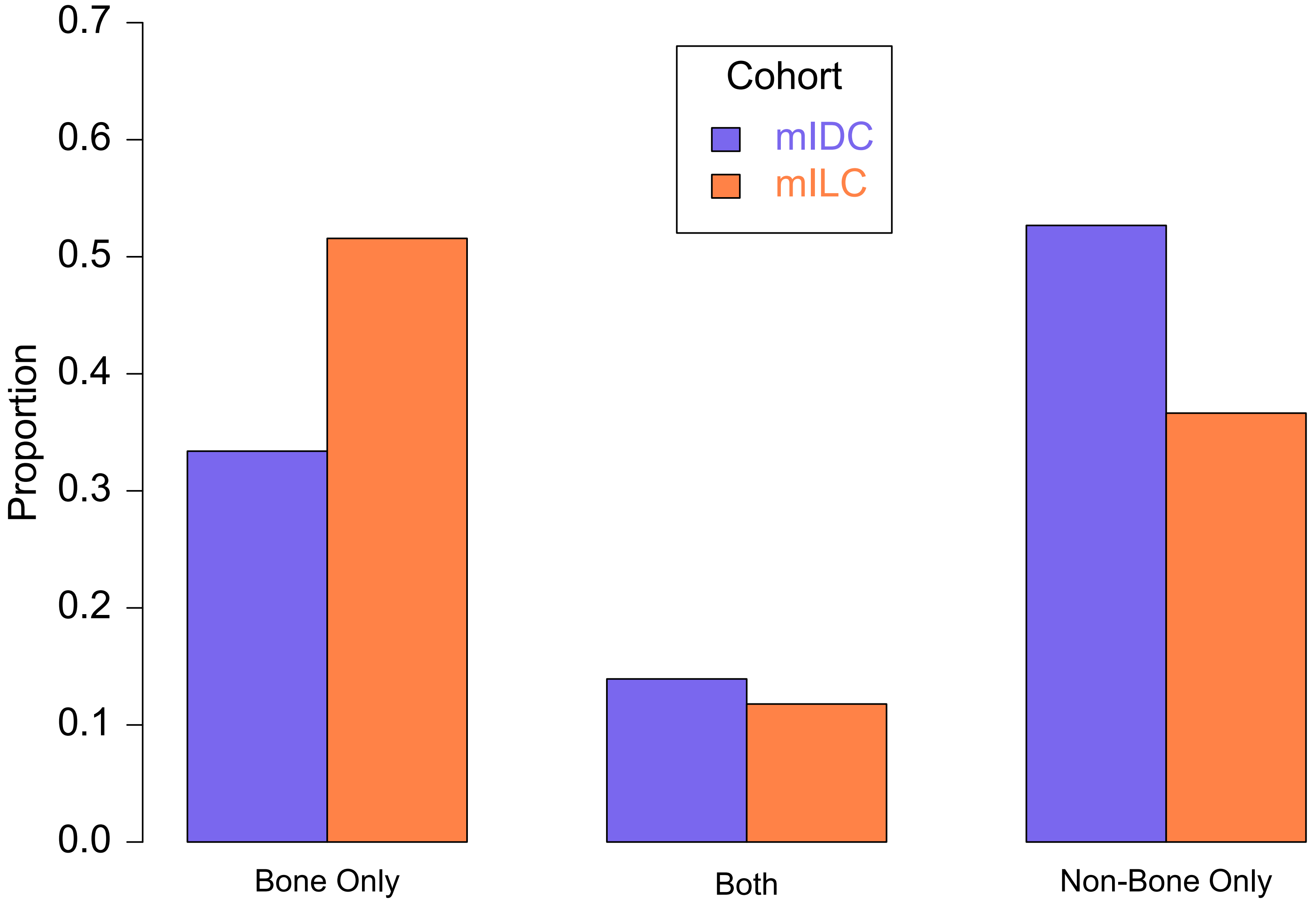

© 2025 Raghavendra AS et al. *JAMA Network Open.*

eFigure 5A: Progression-Free Survival by Histologic Type

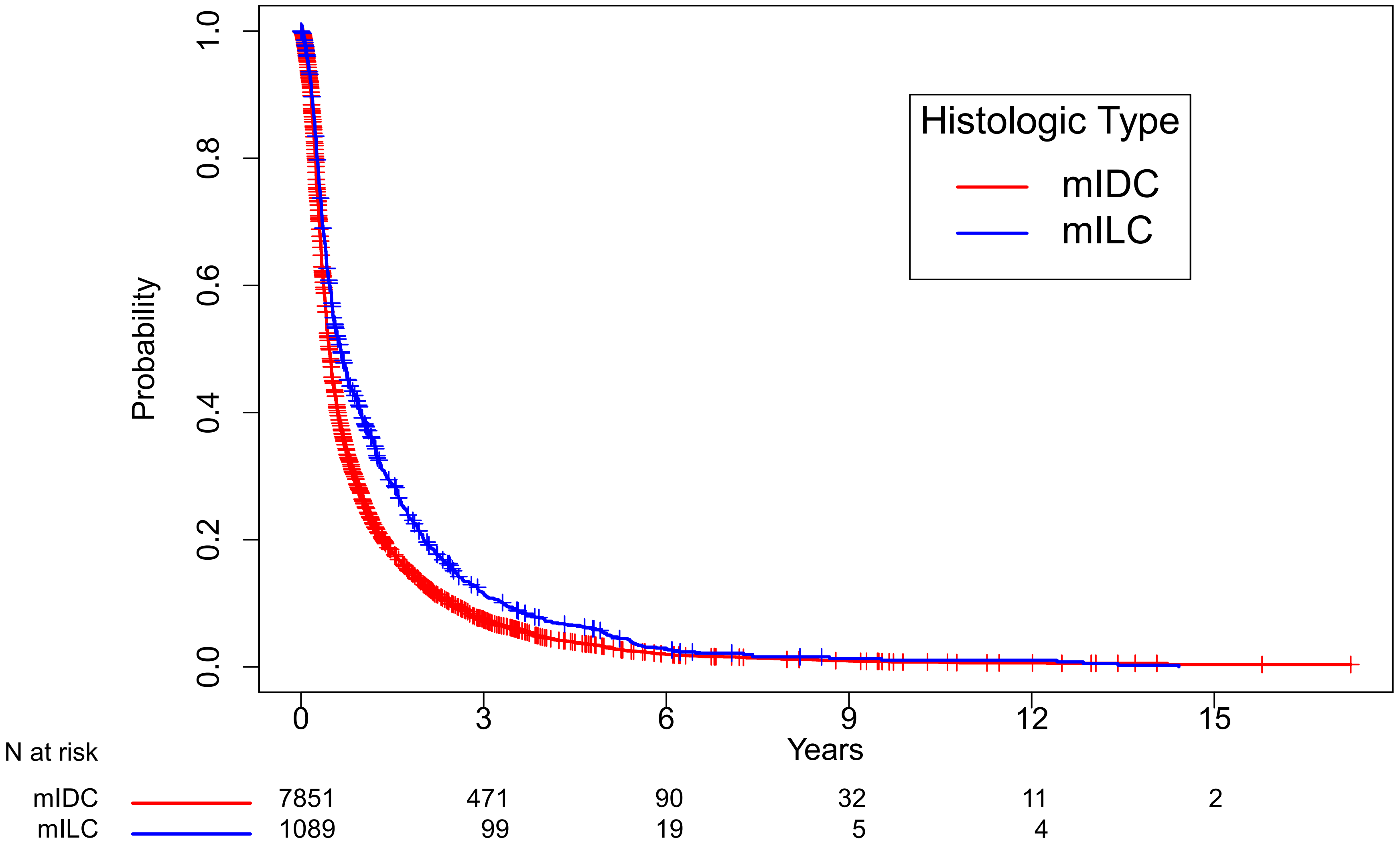

© 2025 Raghavendra AS et al. *JAMA Network Open.*

eFigure 5B: Progression-Free Survival by Histologic Type and Presentation

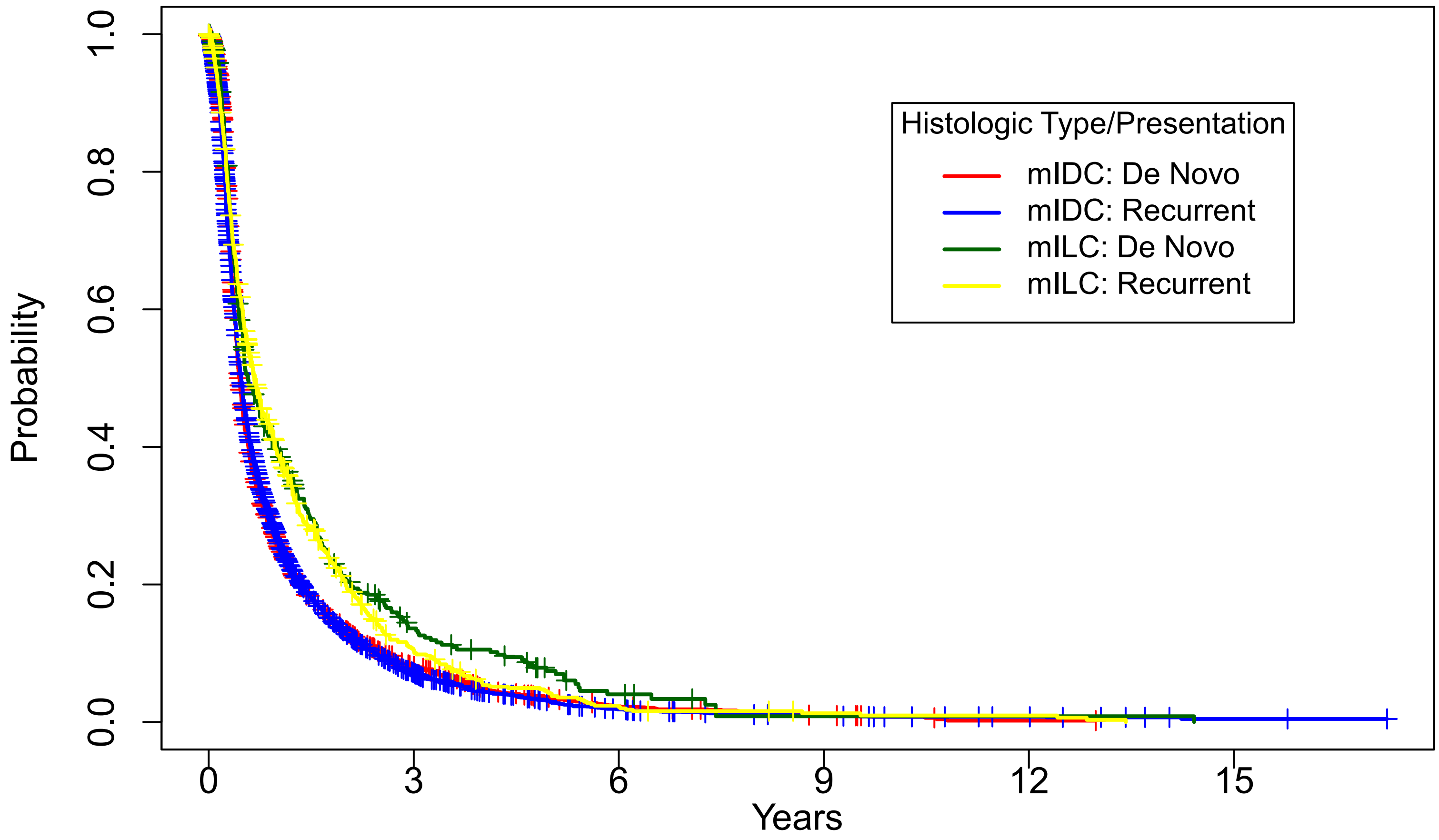

N at risk	0	3	6	9	12	15
mIDC: De Novo	2407	159	32	11	1	
mIDC: Recurrent	5444	312	58	21	10	2
mILC: De Novo	389	40	8	1	1	
mILC: Recurrent	700	59	11	4	3	

© 2025 Raghavendra AS et al. *JAMA Network Open.*

eFigure 5C: Progression-Free Survival by Histologic Type and ER Percent

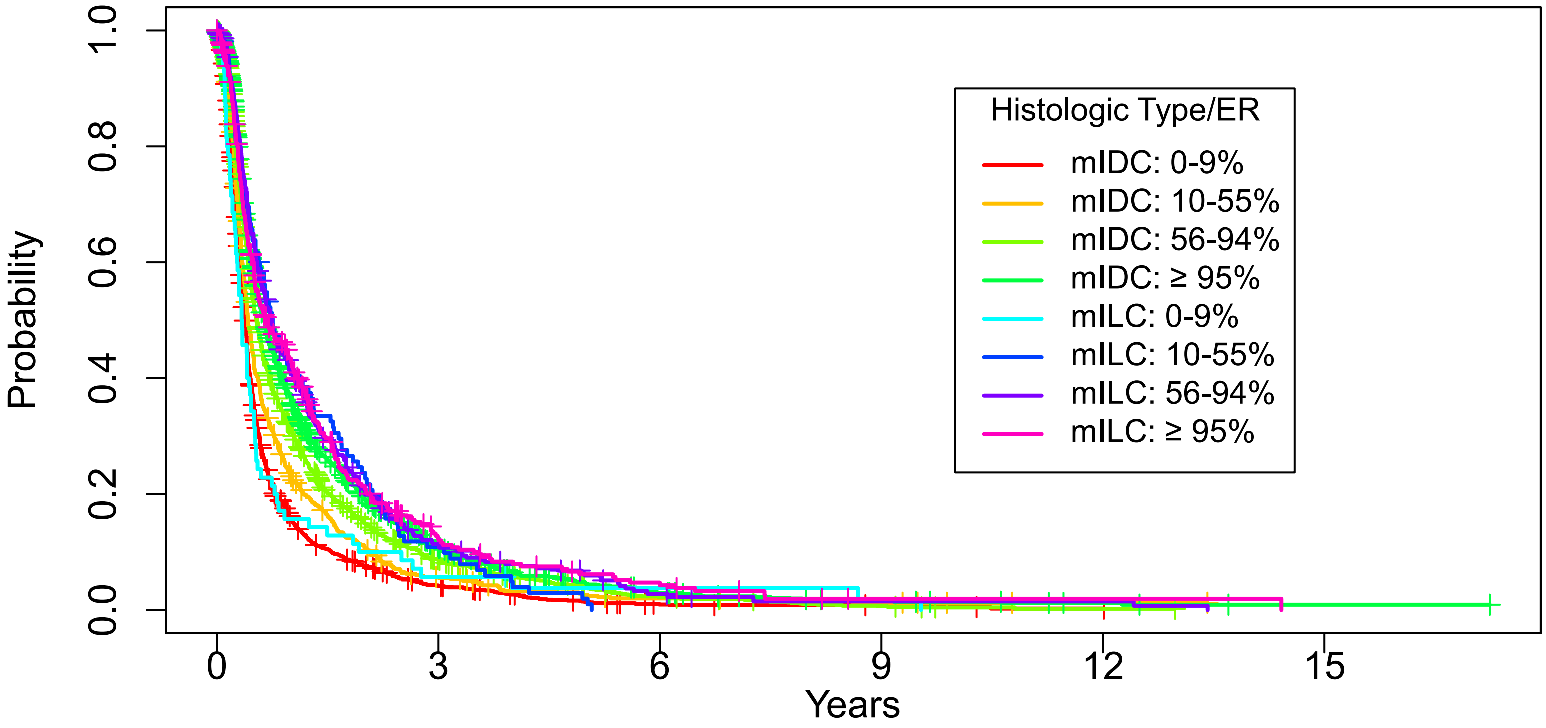

N at risk

	0	3	6	9	12	15
mIDC: 0-9%	1868	67	8	5	1	
mIDC: 10-55%	695	33	9	5	1	
mIDC: 56-94%	1967	138	28	7	1	
mIDC: ≥ 95%	2002	163	33	11	4	1
mILC: 0-9%	70	4	2	1		
mILC: 10-55%	106	11				
mILC: 56-94%	368	34	6	2	2	
mILC: ≥ 95%	367	31	9	1	1	

© 2025 Raghavendra AS et al. *JAMA Network Open.*

eFigure 5D: Progression-Free Survival by Histologic Type and Tumor Grade

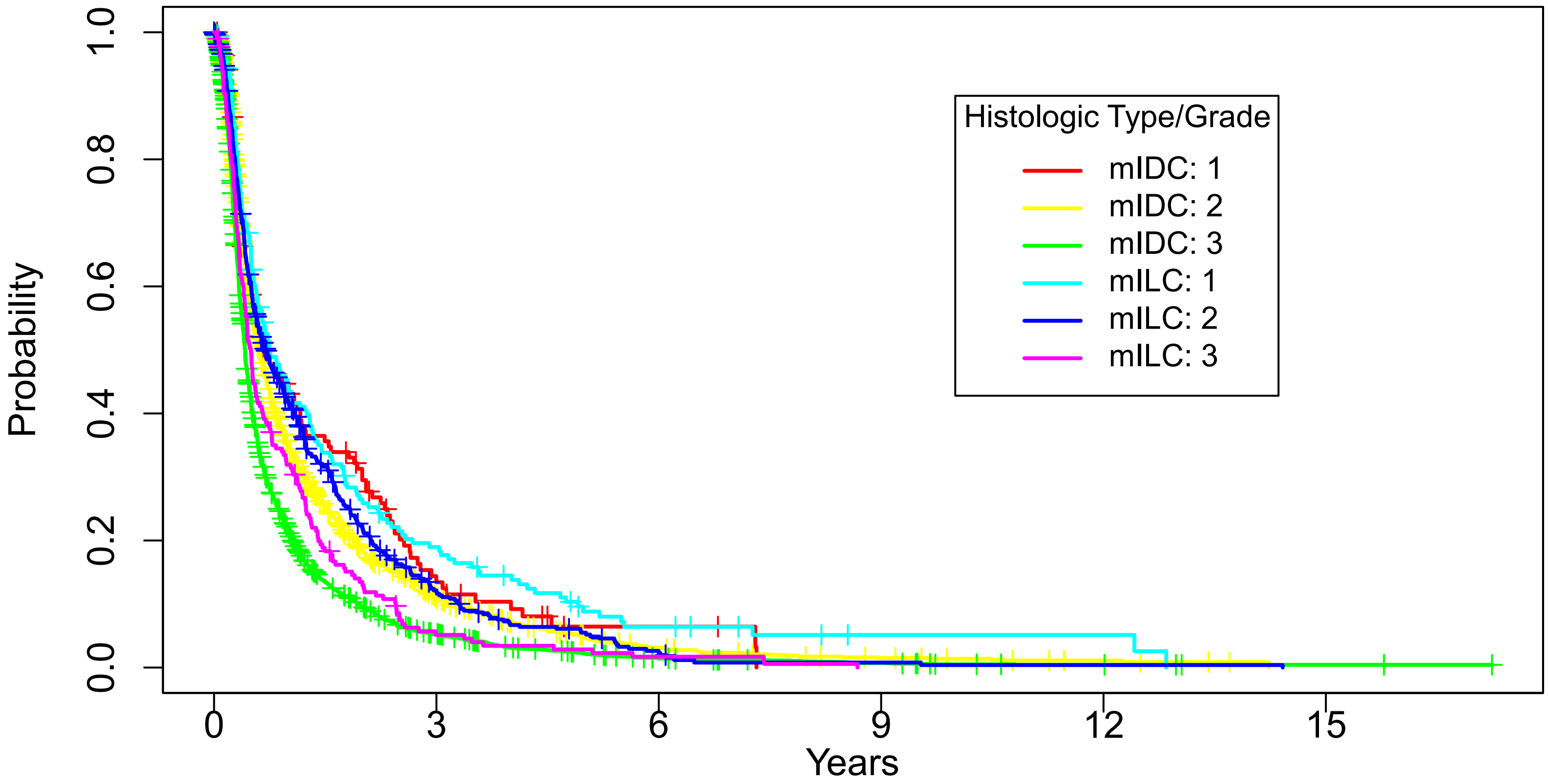

N at risk

	0	3	6	9	12	15
mIDC: 1	141	14	3			
mIDC: 2	2220	192	41	14	5	
mIDC: 3	4485	206	41	17	5	2
mILC: 1	177	30	8	2	2	
mILC: 2	510	46	7	2	1	
mILC: 3	199	9	3			

© 2025 Raghavendra AS et al. *JAMA Network Open.*

eFigure 6: Forest Plot for PFS

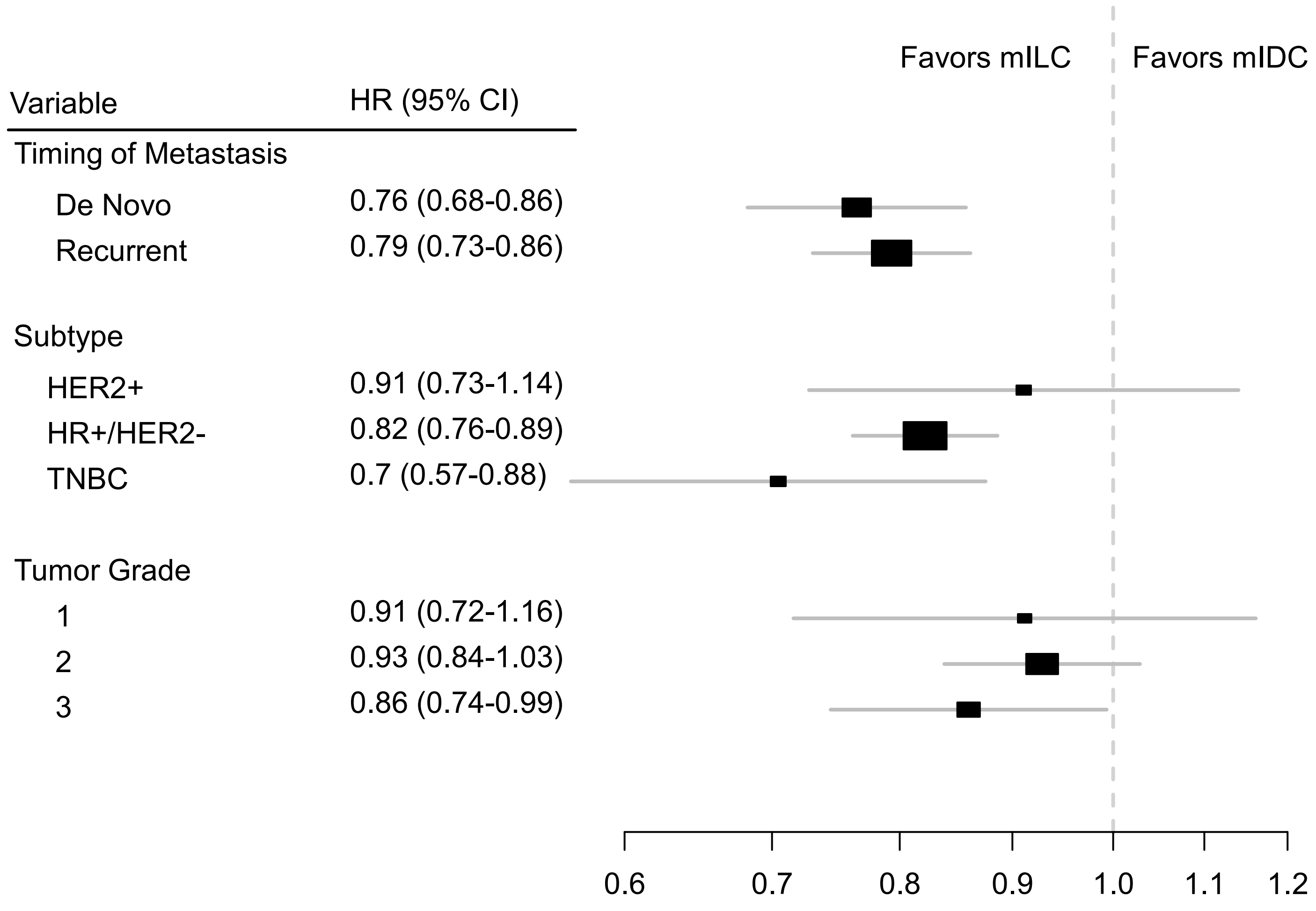

© 2025 Raghavendra AS et al. *JAMA Network Open.*

HR (95% CI)

eFigure 7A: Overall Survival by Histologic Type

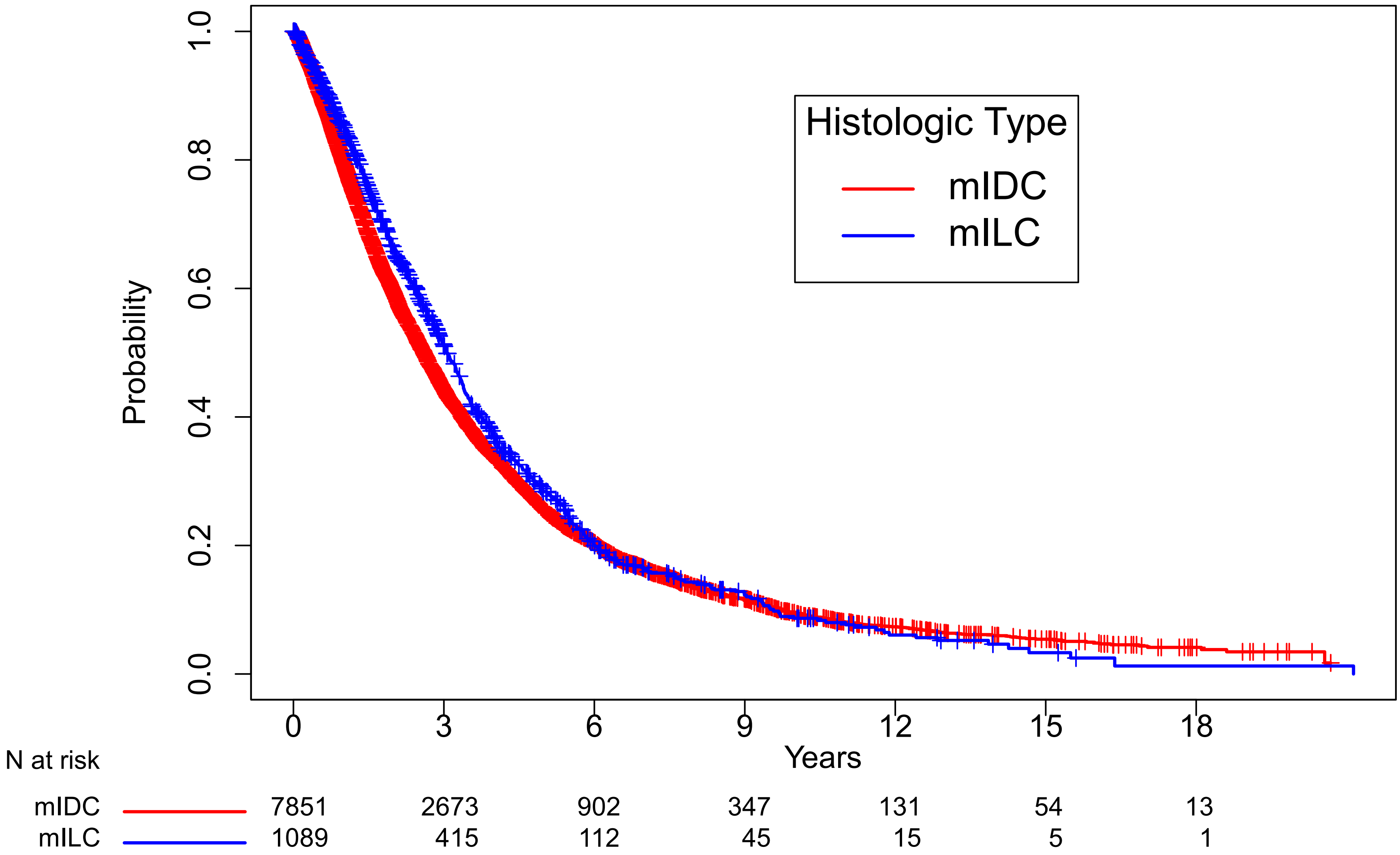

© 2025 Raghavendra AS et al. *JAMA Network Open.*

eFigure 7B: Overall Survival by Histologic Type and Presentation

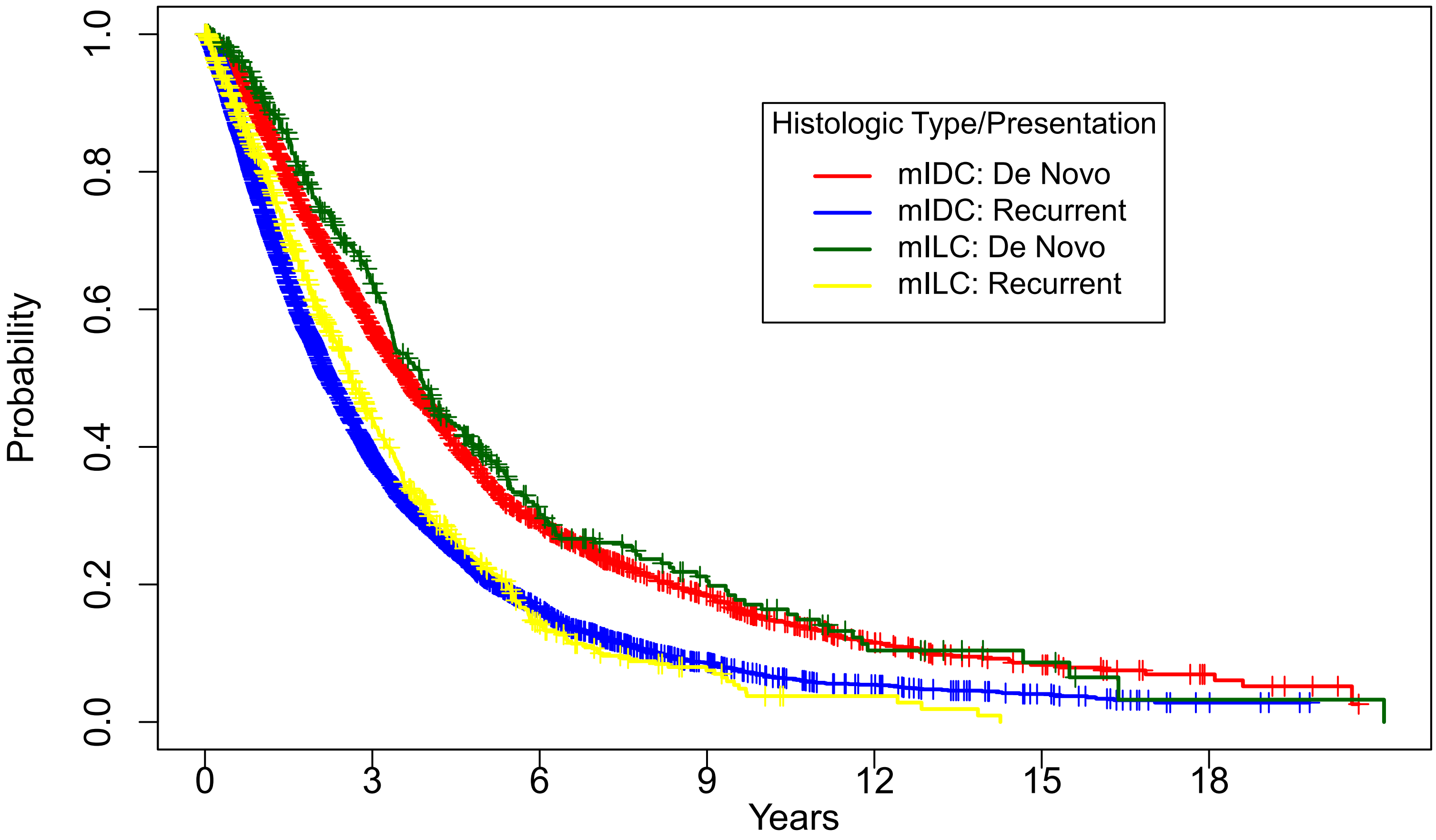

N at risk

	0	3	6	9	12	15	18
mIDC: De Novo	2407	1084	407	173	60	26	8
mIDC: Recurrent	5444	1589	495	174	71	28	5
mILC: De Novo	389	191	63	30	11	5	1
mILC: Recurrent	700	224	49	15	4		

© 2025 Raghavendra AS et al. *JAMA Network Open.*

eFigure 7C: Overall Survival by Histologic Type and ER Percent

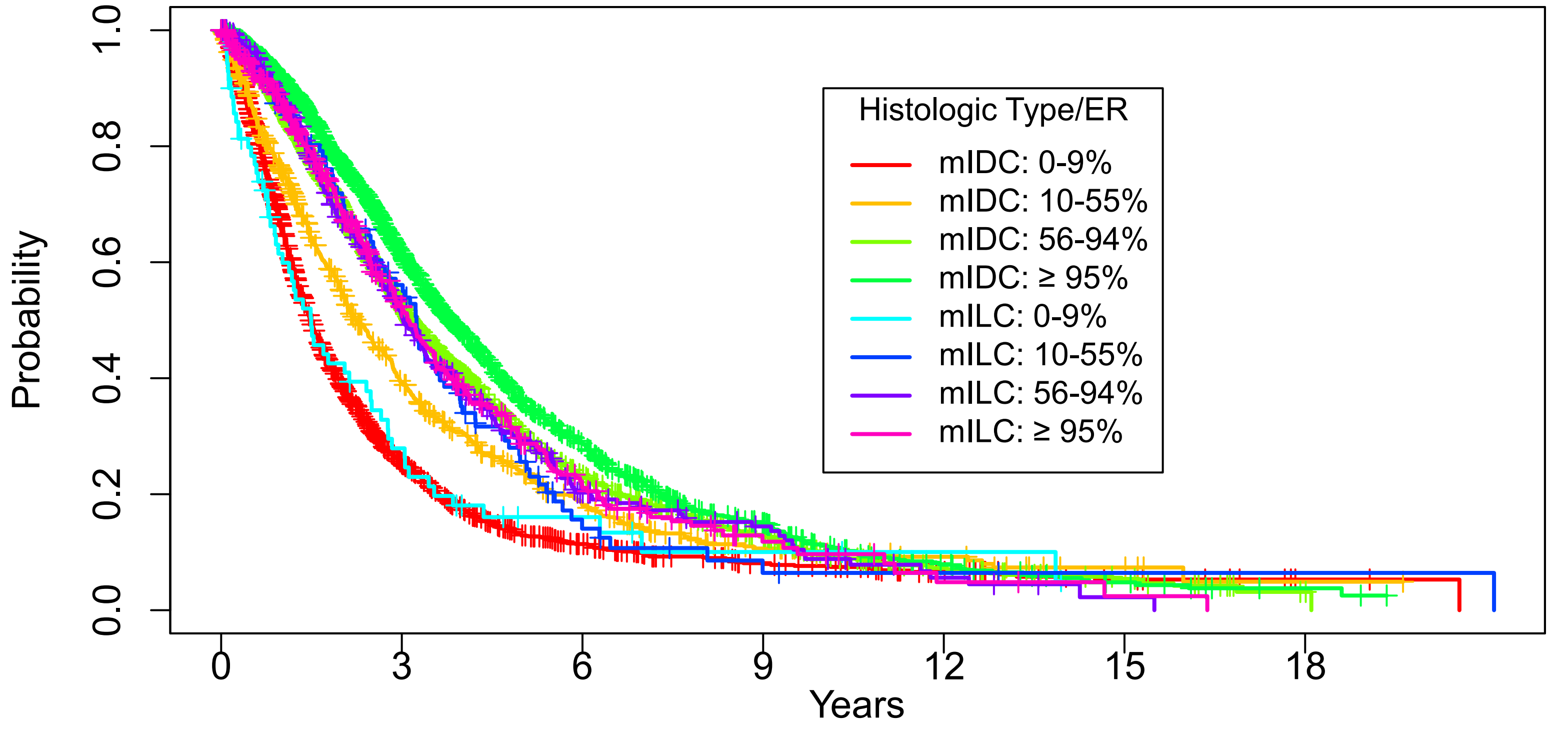

N at risk	0	3	6	9	12	15	18
mIDC: 0-9%	1868	361	112	52	23	10	2
mIDC: 10-55%	695	223	80	28	16	6	1
mIDC: 56-94%	1967	805	271	107	32	15	2
mIDC: ≥ 95%	2002	884	287	97	31	11	3
mILC: 0-9%	70	17	6	3	2		
mILC: 10-55%	106	52	9	3	2	1	1
mILC: 56-94%	368	143	38	18	5	1	
mILC: ≥ 95%	367	128	37	11	3	1	

© 2025 Raghavendra AS et al. *JAMA Network Open.*

eFigure 7D: Overall Survival by Histologic Type and Tumor Grade

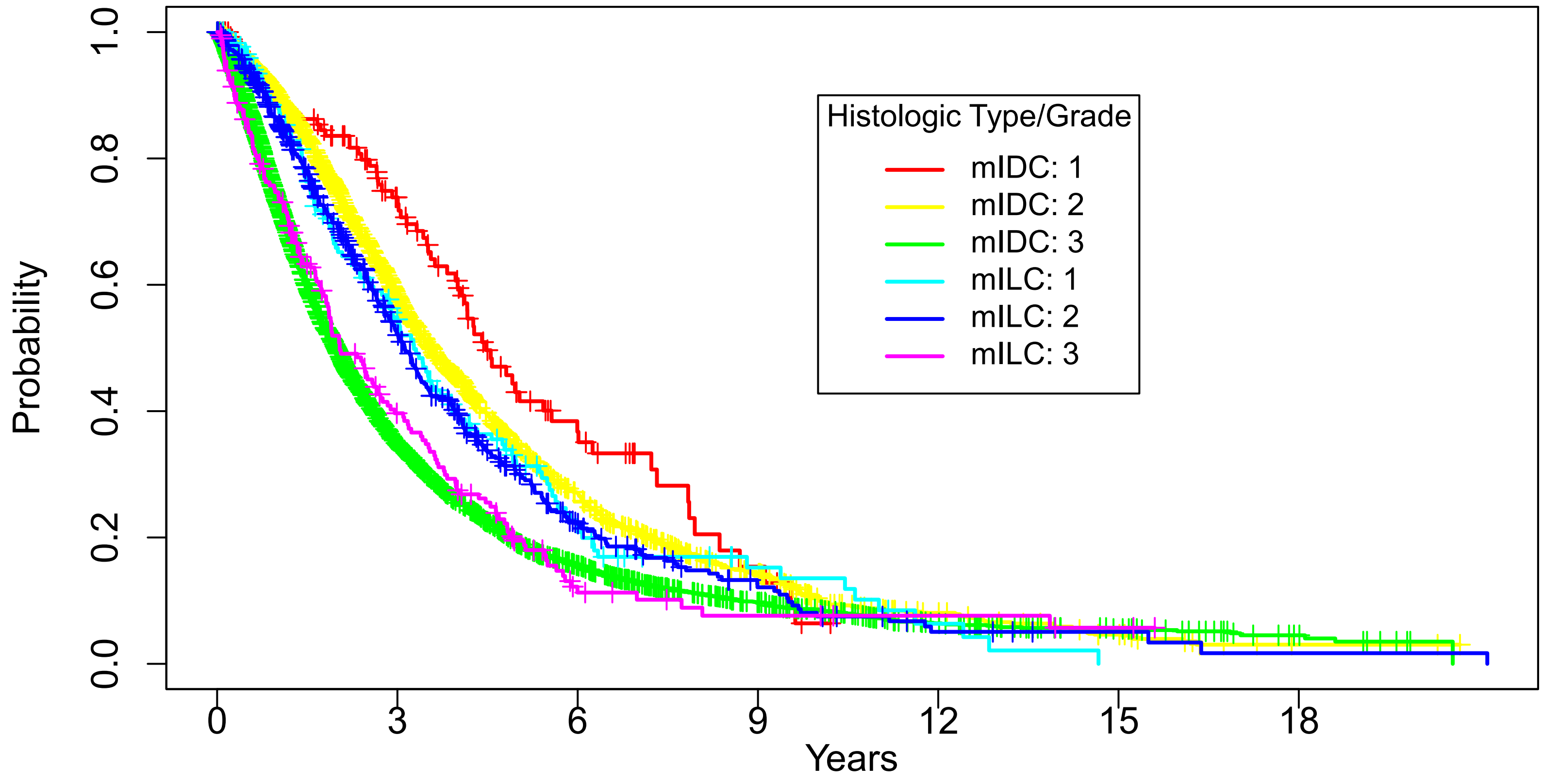

N at risk

	0	3	6	9	12	15	18
mIDC: 1	141	70	22	6			
mIDC: 2	2220	997	343	128	41	16	2
mIDC: 3	4485	1282	442	186	73	34	10
mILC: 1	177	75	22	9	3		
mILC: 2	510	200	59	21	6	3	1
mILC: 3	199	65	12	6	4	2	

© 2025 Raghavendra AS et al. *JAMA Network Open.*

eFigure 8: Forest Plot for OS

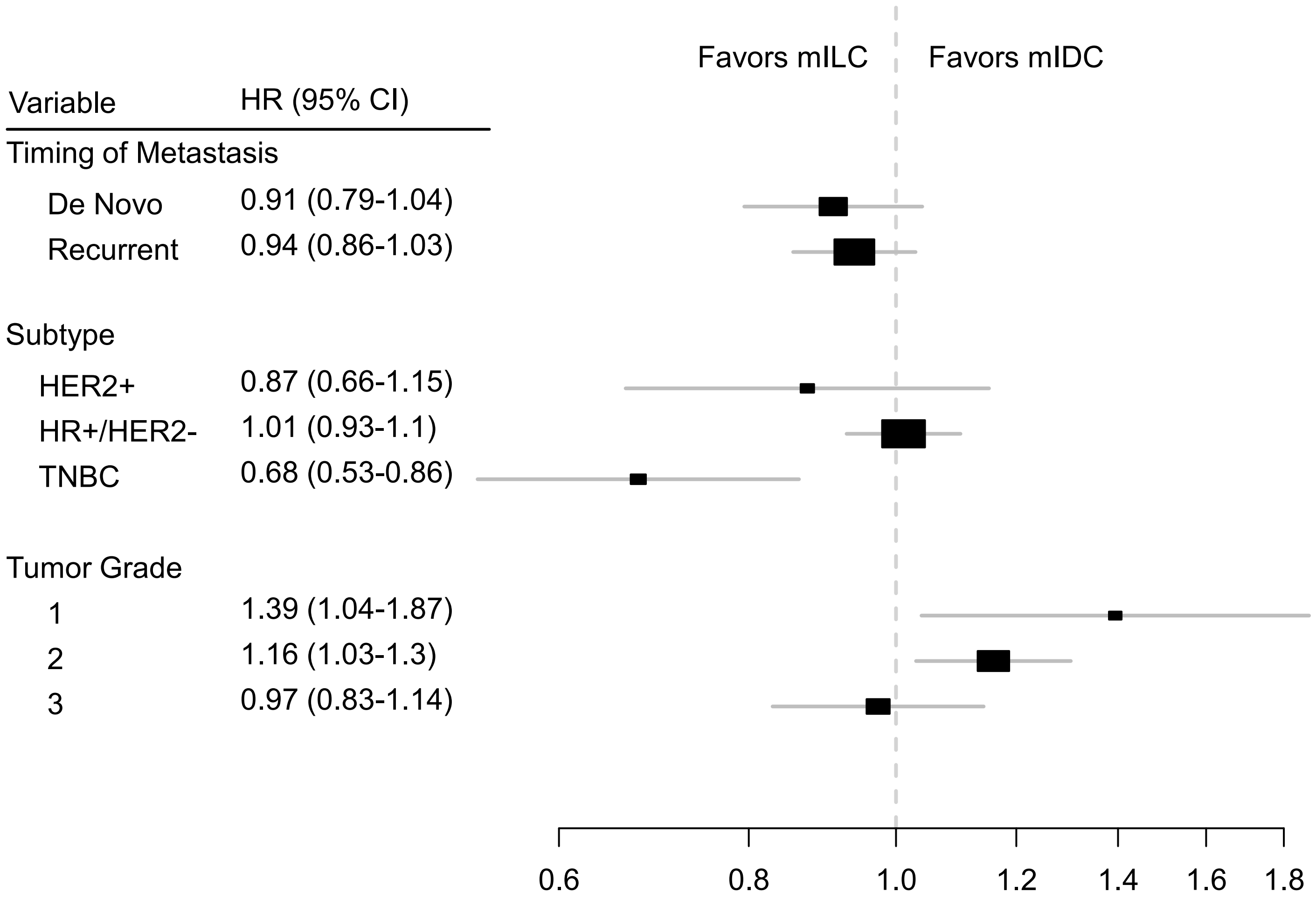

© 2025 Raghavendra AS et al. *JAMA Network Open.*

HR (95% CI)

eFigure 9A: Disease-Free Interval by Histologic Type

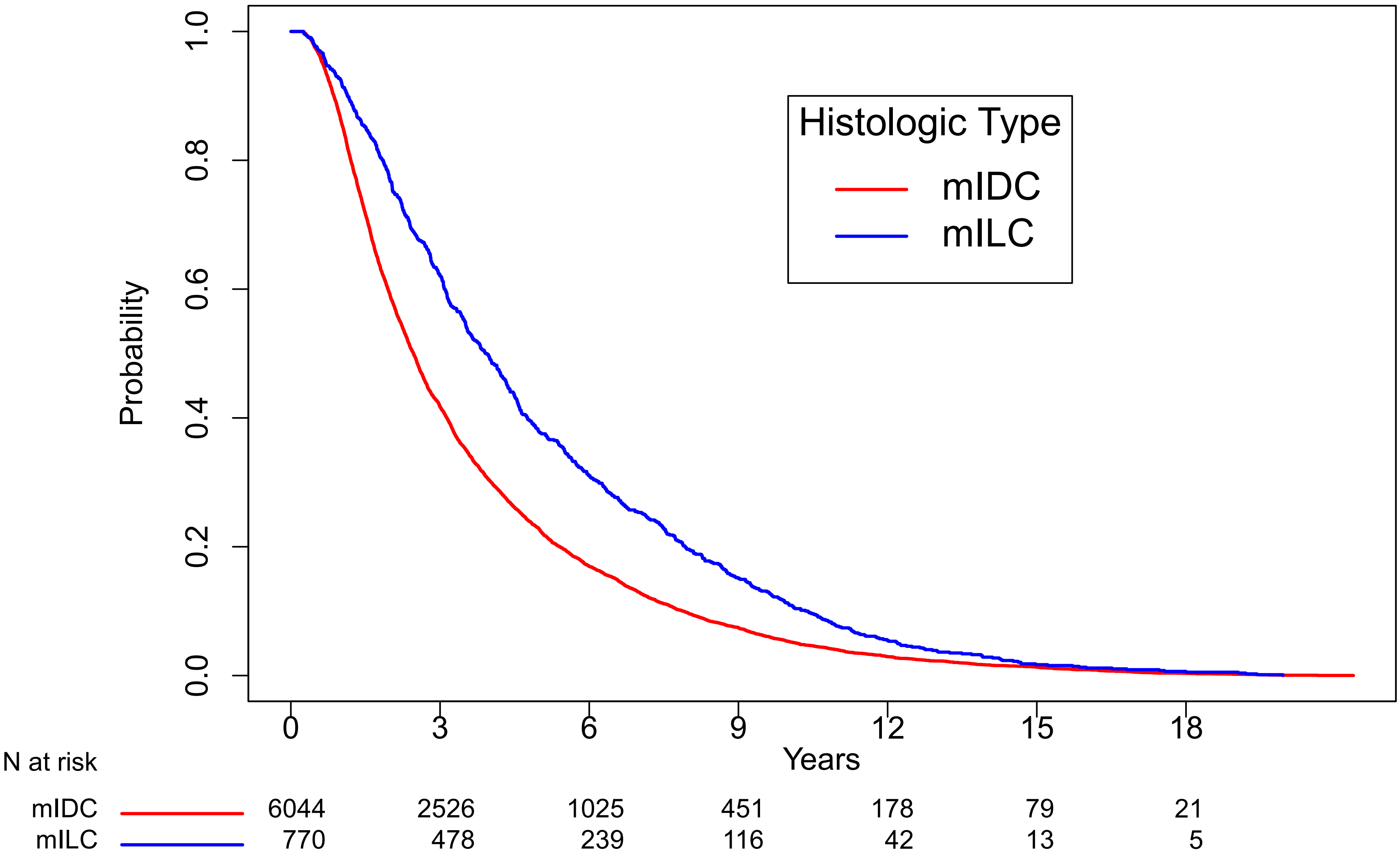

© 2025 Raghavendra AS et al. *JAMA Network Open.*

eFigure 9B: Disease-Free Interval by Histologic Type and Grade

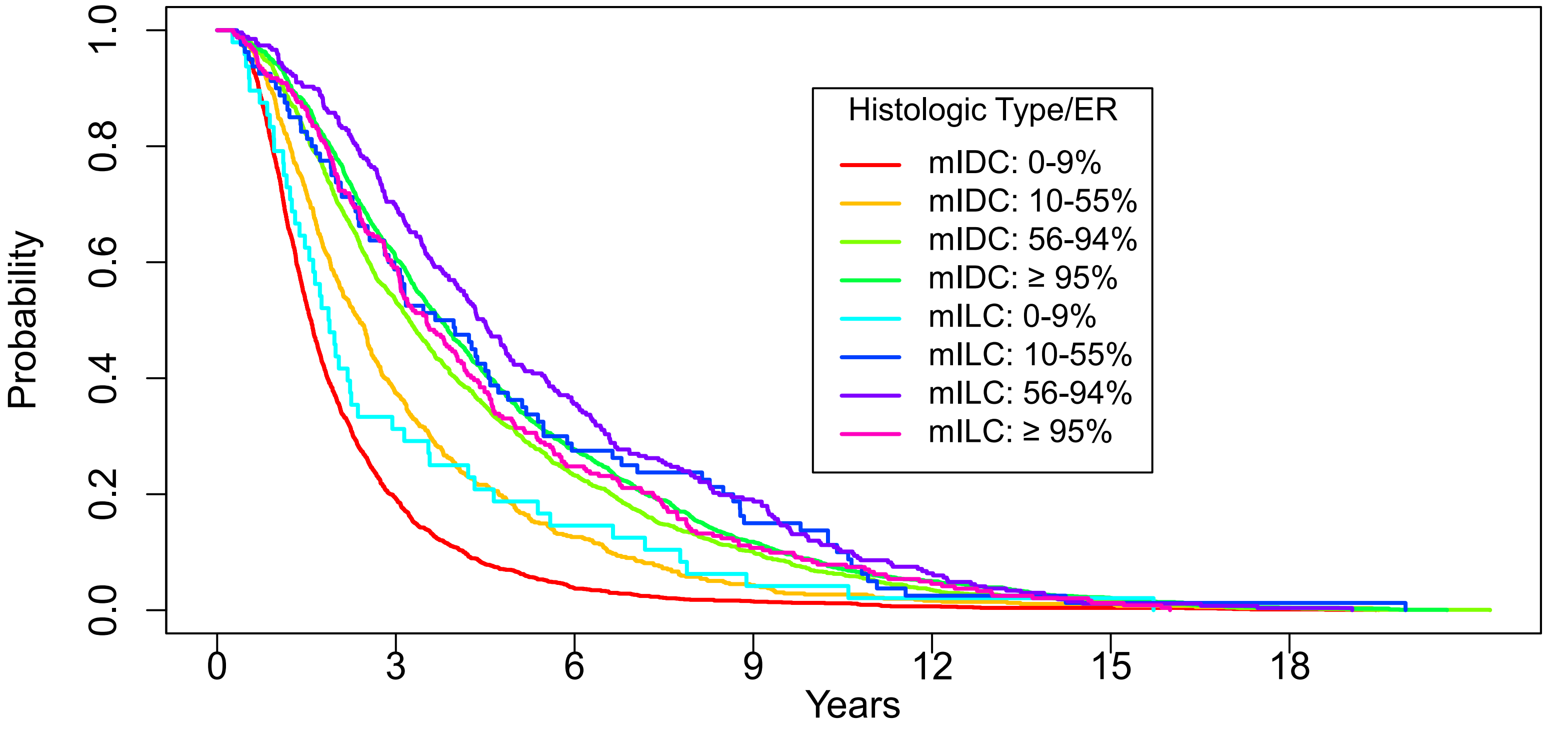

N at risk

mIDC: 0-9%	1508	291	57	23	10	6	2
mIDC: 10-55%	555	208	70	24	9	6	2
mIDC: 56-94%	1484	792	345	150	52	23	6
mIDC: ≥ 95%	1424	864	394	168	71	30	6
mILC: 0-9%	48	15	7	2	1	1	
mILC: 10-55%	80	47	22	12	2	1	1
mILC: 56-94%	267	186	95	50	17	3	1
mILC: ≥ 95%	242	143	60	26	11	3	

© 2025 Raghavendra AS et al. *JAMA Network Open.*

eFigure 9C: Disease-Free Interval by Histologic Type and Tumor Grade

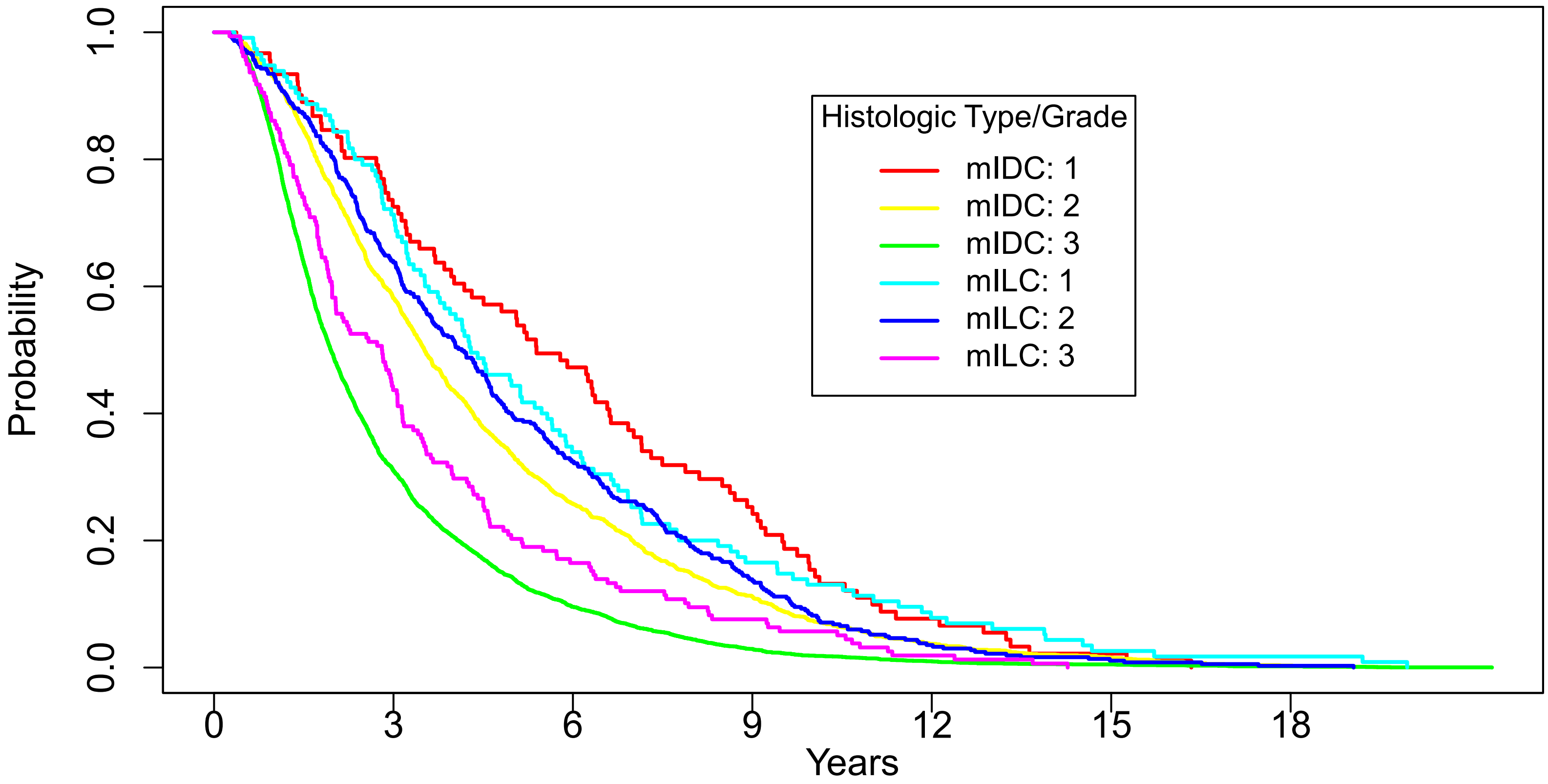

N at risk

mIDC: 1	91	66	43	23	7	2	
mIDC: 2	1609	936	414	180	60	25	5
mIDC: 3	3612	1121	345	106	36	18	7
mILC: 1	115	82	39	19	9	3	2
mILC: 2	367	234	119	50	13	4	1
mILC: 3	158	69	26	12	3		

© 2025 Raghavendra AS et al. *JAMA Network Open.*

eFigure 10: Forest Plot for DFI

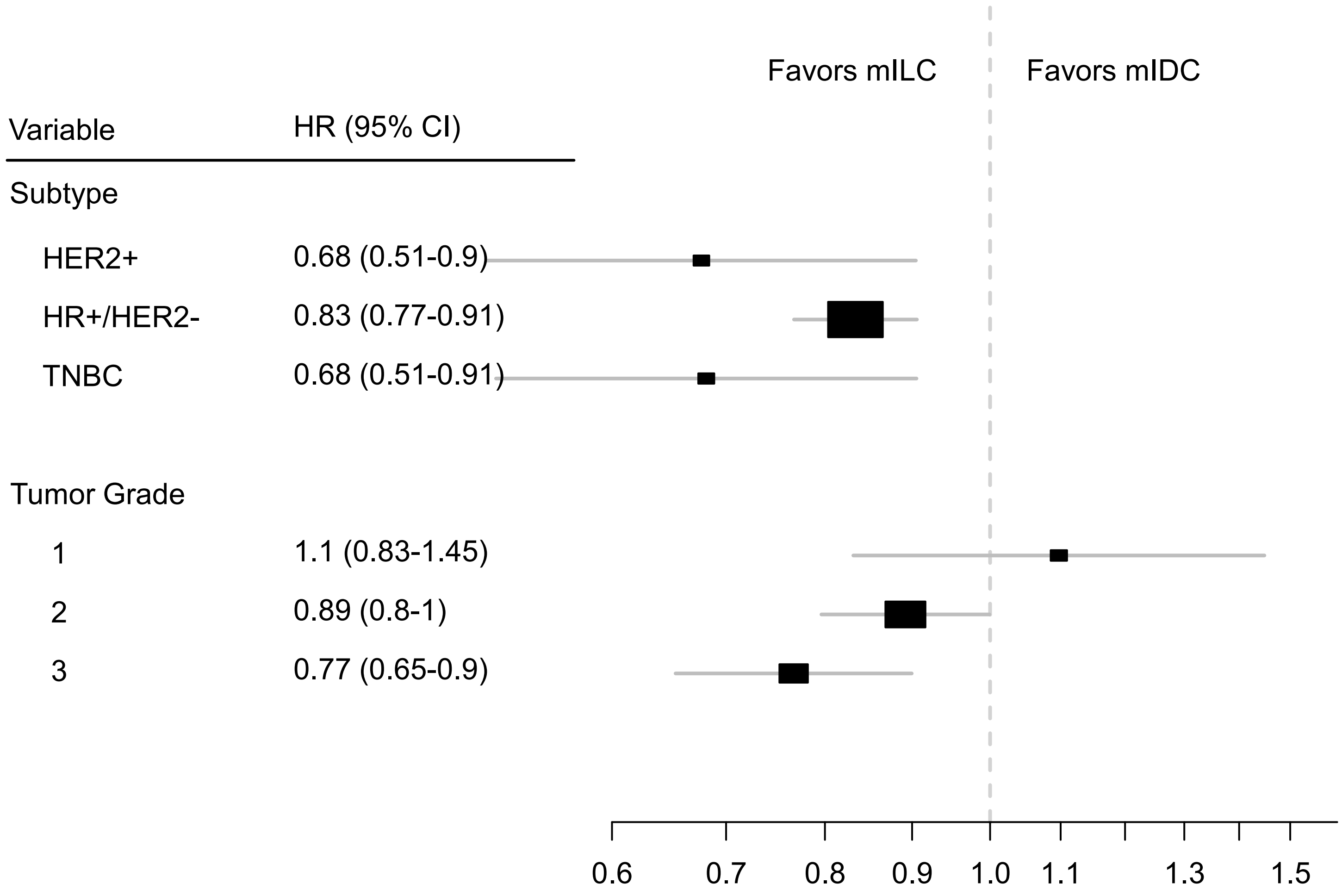

© 2025 Raghavendra AS et al. *JAMA Network Open.*

HR (95% CI)

eFigure 11. Distribution of key genomic alterations according to histologic type across subtypes

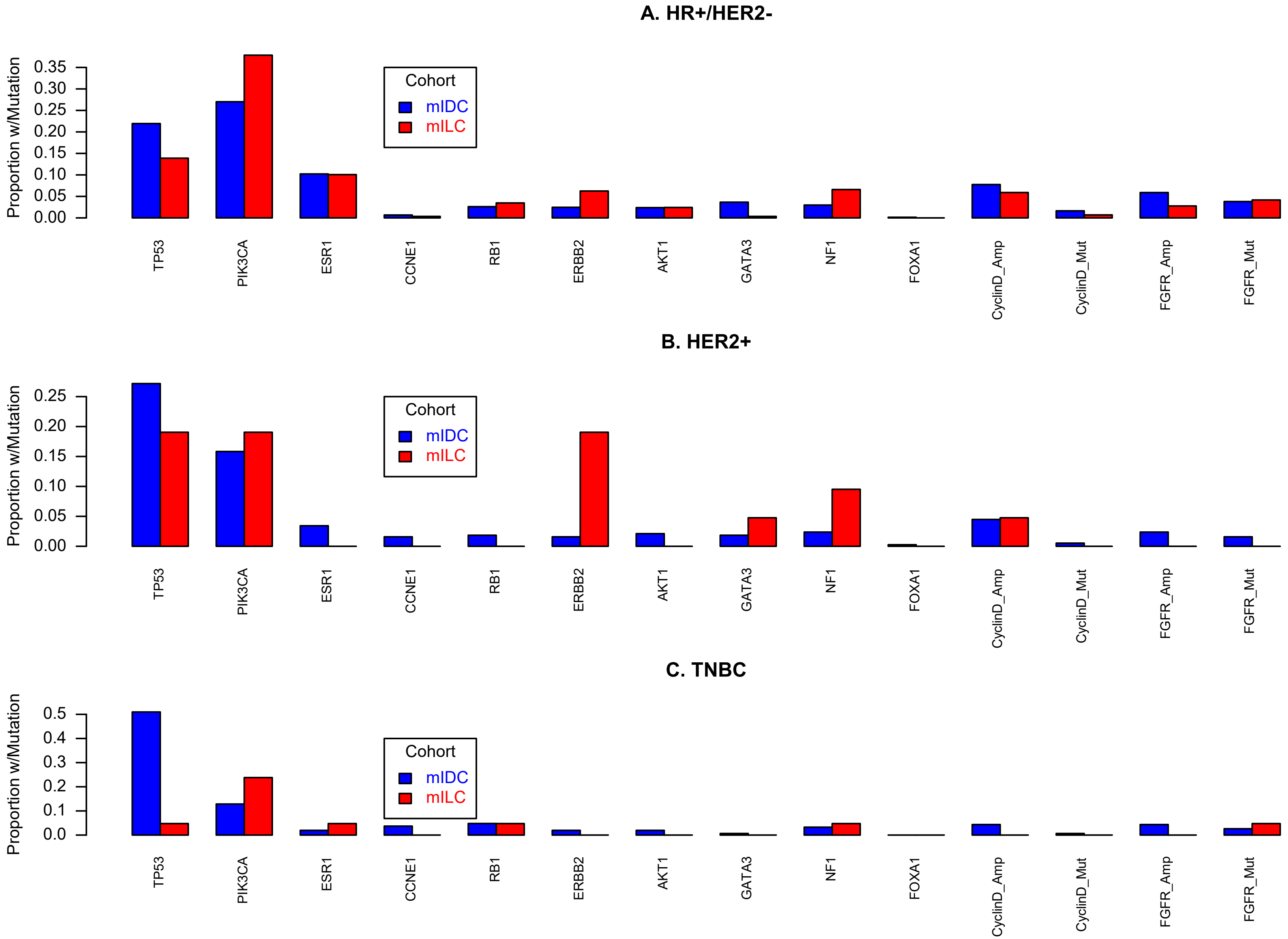

© 2025 Raghavendra AS et al. *JAMA Network Open.*
