# Supplementary material for: Clinical Characteristics and Survival Outcomes of Metastatic Invasive Lobular and Ductal Carcinoma
Source: JAMA Netw Open. 2025 Apr 28;8(4):e251888. doi: 10.1001/jamanetworkopen.2025.1888 (PMC12038507; doi:10.1001/jamanetworkopen.2025.1888)
Supplement: Supplement 2. — Data Sharing Statement [file jamanetwopen-e251888-s002.pdf]

Data Sharing Statement

Raghavendra. Clinical Characteristics and Survival Outcomes of Metastatic Invasive Lobular and Ductal Carcinoma. *JAMA Netw Open*. Published March 25, 2025. doi:10.1001/jamanetworkopen.2025.1888

Data
Data available: No

Additional Information
Explanation for why data not available: The data that support the findings of this study are available on reasonable request to the corresponding author. The data are not publicly available to protect the privacy of the study participants.
